# Liver transplantation vs liver resection in HCC: promoting extensive collaborative research through a survival meta-analysis of meta-analyses

**DOI:** 10.3389/fonc.2024.1366607

**Published:** 2024-03-18

**Authors:** Alessandro Martinino, Angela Bucaro, Francesca Cardella, Ishaan Wazir, Francesco Frongillo, Francesco Ardito, Francesco Giovinazzo

**Affiliations:** ^1^ Department of Surgery, Duke University, Durham, NC, United States; ^2^ General Surgery and Liver Transplant Unit, Fondazione Policlinico Universitario Agostino Gemelli IRCCS, Rome, Italy; ^3^ Surgical Oncology of Gastrointestinal Tract Unit, Vanvitelli University, Naples, Italy; ^4^ Vardhaman Mahavir Medical College & Safdarjung Hospital, New Delhi, India; ^5^ Hepatobilairy and General Surgery Unit, Fondazione Policlinico Universitario Agostino Gemelli IRCCS, Rome, Italy

**Keywords:** liver transplantation, liver resection, hepatocellular carcinoma, survival, meta-analysis

## Abstract

**Background:**

HCC is a major global health concern, necessitating effective treatment strategies. This study conducts a meta-analysis of meta-analyses comparing liver resection (LR) and liver transplantation (LT) for HCC.

**Methods:**

The systematic review included meta-analyses comparing liver resection vs. liver transplantation in HCC, following PRISMA guidelines. Primary outcomes included 5-year overall survival (OS) and disease-free survival (DFS). AMSTAR-2 assessed study quality. Citation matrix and hierarchical clustering validated the consistency of the included studies.

**Results:**

A search identified 10 meta-analyses for inclusion. The median Pearson correlation coefficient for citations was 0.59 (IQR 0.41-0.65). LT showed better 5-year survival and disease-free survival in all HCC (OR): 0.79; 95% CI: 0.67-0.93, I^2:57% and OR: 0.44; 95% CI: 0.25-0.75, I^2:96%). Five-year survival in early HCC and ITT was 0.63 (95% CI: 0.50-0.78, I^2:0%) and 0.60 (95% CI: 0.39-0.92, I^2:0%). Salvage LT vs. Primary LT did not differ between 5-year survival and disease-free survival (OR: 0.62; 95% CI: 0.33-1.15, I^2:0% and 0.93; 95% CI: 0.82-1.04, I^2:0%).

**Conclusion:**

Overall, the study underscores the superior survival outcomes associated with LT over LR in HCC treatment, supported by comprehensive meta-analysis and clustering analysis. There was no difference in survival or recurrence rate between salvage LT and primary LT. Therefore, considering the organ shortage, HCC can be resected and transplanted in case of recurrence.

## Introduction

Hepatocellular carcinoma (HCC), with 782000 cases diagnosed and 746 000 deaths in 2012 and an age-adjusted worldwide incidence of 10·1 cases per 100 000 person-years (1), is the sixth most common cancer and the third-leading cause of cancer-related mortality in the world (1, 2).

HCC usually develops in the setting of chronic liver diseases, such as cirrhosis, infections like hepatitis B or C, non-alcoholic fatty liver disease, or alcohol-related liver disease (1–3). Most HCCs (80%) occur in sub-Saharan Africa and eastern Asia, where the main risk factors are chronic hepatitis B and aflatoxin B1 exposure. Instead, in the USA, Europe, and Japan, hepatitis C is the leading risk factor, together with excessive alcohol intake (1, 4, 5).

The management of HCC depends on several factors, including the size and number of tumours, the underlying liver function, and the patient’s overall health status (6, 7). Liver resection (LR) and transplantation (LT) are the most effective curative treatments for HCC, with promising outcomes in survival and disease-free survival (DFS) (1, 8–10). In patients without clinically significant portal hypertension (CSPH), compensated liver function, and early HCC stages, LR achieves 70% 5-year survival in HCC. However, the survival rate decreases by 50% when those adverse factors are present (1). On the other hand, 5-year survival in HCC after LT is more than 70% with a recurrence rate of less than 10–15% (1) (11). However, the choice of the two treatments is also limited by the availability of donor organs. Therefore, choosing between LT and LR for HCC in several cases is still controversial (7, 10).

As robust evidence is missing with contrasting results, the objective of the present study was to perform a survival meta-analysis of meta-analyses to compare LT and LR in HCC. The primary outcomes were 5-year overall and disease-free survival after the two different types of treatment.

## Methods

The systematic review and meta-analysis were conducted according to the Preferred Reporting Items for Systematic Reviews and Meta-Analyses (PRISMA) guidelines.

A computerised search of PubMed, Scopus and Cochrane Library was carried out. Reference lists of all obtained and relevant articles were screened manually and cross-referenced to identify any additional studies. Articles published from the time of inception to June 2023 were included. An advanced search was performed using the following terms: [(transplant) OR (transplantation)] AND (hepatocellular) OR (HCC) OR (liver cancer).

### Outcomes of interest

The primary outcomes were 5-year graft overall (OS) and disease-free survival (DFS) in liver resection vs. liver transplantation in all HCCs. The secondary outcomes were OS and DSF in early HCC, Intention to treat, and salvage liver transplantation for HCC.

### Inclusion criteria

The systematic review included meta-analyses comparing liver resection vs. liver transplantation in HCC and reporting the primary and secondary outcomes. Abstracts, letters, comments, editorials and expert opinions, unpublished articles and abstracts, reviews without original data, and case reports were excluded from the analysis. Studies were included only when reporting the number or the rate of events (deaths or recurrences). Two reviewers (AM and IW) independently screened the titles and abstracts of all retrieved articles. The full texts of articles that could fulfil the inclusion criteria were obtained and checked for eligibility.

### Internal validity (methodological quality)

The internal validity of the meta-analyses was assessed by the Assessment of Multiple Systematic Reviews 2 (AMSTAR-2) method. AMSTAR is a standardised and reliable method for assessing the quality of systematic reviews that include randomised or non-randomised studies of healthcare interventions, or both. FG and FC completed the AMSTAR proforma for all included reviews, and discrepancies were discussed to reach a consensus. Studies were, finally, classified on the level of quality through the online tool calculator (12).

### Cytation matrix and dendrogram analysis

A sample citation matrix was created by measuring the primary overlap of every included study (**Supplementary Table 1**), and the Pearson correlation coefficient (r) was calculated. r was visualised through a heatmap. A hierarchical cluster analysis of the r was visualised through a dendrogram clusterisation and a silhouette analysis used to identify the number of clusters (13).

### Data analysis

The results of the meta-analyses were combined using a summary meta-analysis model for odds ratios (OR) and hazard ratio (HR) with 95% confidence intervals.

The fixed-effect method was used to combine the results without statistically significant heterogeneity. The random-effect method was used when heterogeneity was confirmed (p ≤0·10). Potential publication bias was investigated by funnel plot. Egger’s and Begger’s tests were used to assess funnel plot asymmetry and biases [12], and Makaskill’s test was used to quantify the bias (14). P <0·05 (two-tailed) was considered to indicate statistical significance [13]. Trim-and-fill method was used to adjust for the publication biases.

The meta-analysis of meta-analyses and hierarchical analysis was performed using the R software suite (v3.4.0, https://www.R-project.org). Statistical heterogeneity between metanalysis was evaluated by χ2 and I2, with significance set at p ≤0,10 (14–16).

## Results

### Literature search

**Figure 1:**
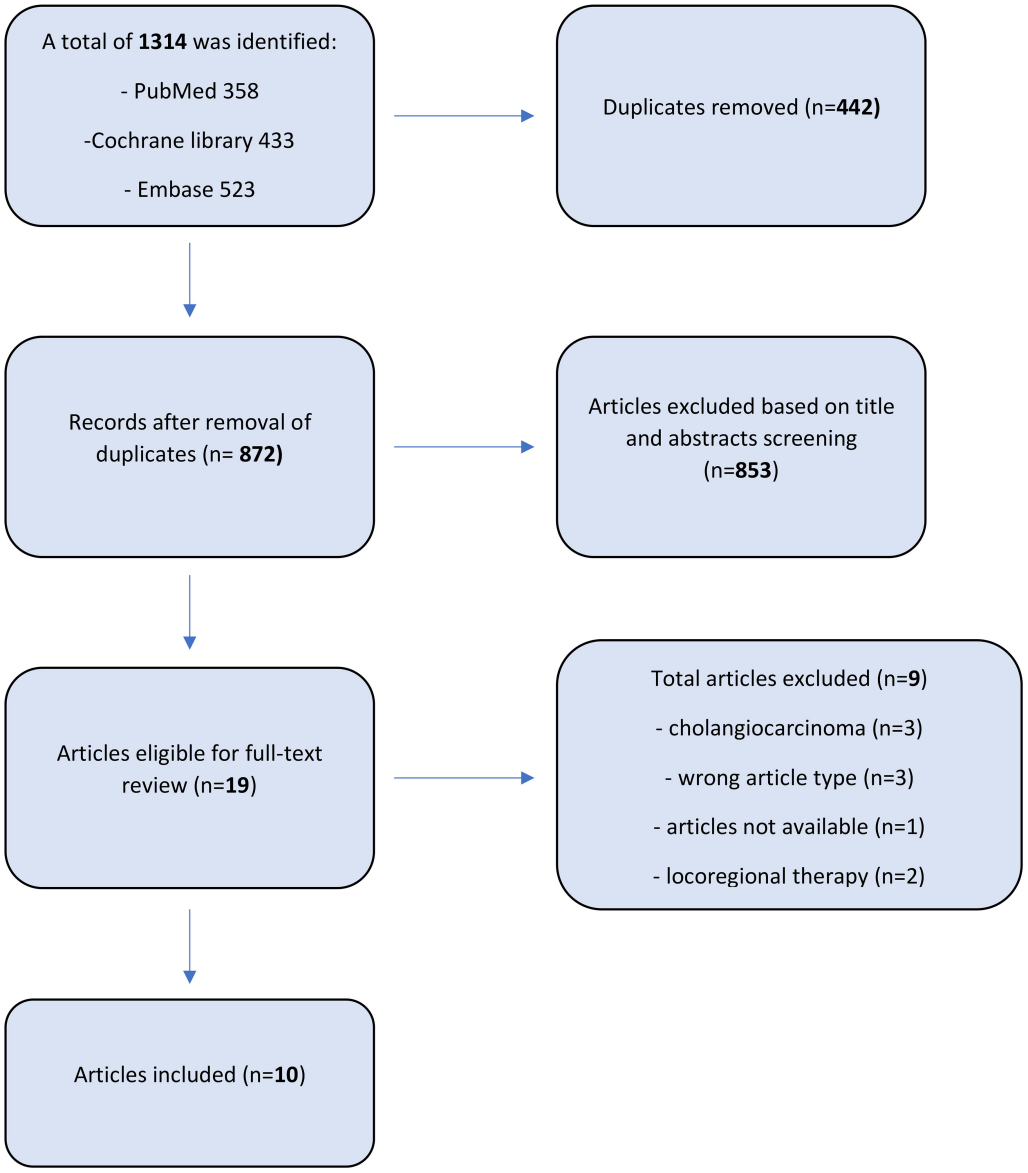
PRISMA flow diagram.

The PRISMA flow diagram reports the number of studies screened, assessed, and excluded (**Figure 1**). 19 full-text articles were assessed for eligibility, and 10 meta-analyses comparing an overall 105 studies were included in the umbrella review (11, 17–25). The characteristics of the included meta-analyses are shown in **Table 1**.

**Table 1:** Included studies.

Authors	Journal	Publication Year	Range of Years of Included Studies	No. of Primary Studies	No. of Retrospective Study	Finding Direction
Dhir et al. (17)	HPB	2012	1990-2011	10	10	LT
Rahman et al. (11)	J Gastrointest Surg	2012	2000-2012	9	9	LT
Li et al. (19)	World J Gastroenterol	2012	1996-2011	11	11	LT
Zheng et al. (25)	Transplantation	2014	Inception to 8 March 2013	62	not specified	LT
Proneth et al. (22)	Ann Surg Oncol	2014	1990-2013	7	7	LT
Xu et al. (24)	Hepatobiliary Pancreat Dis Int	2014	1990-2012	17	17	LT
Menahem et al. (21)	Liver Transplantation	2017	Inception to 8 March 2015	9	9	No differences
Schoenberg et al. (23)	Medicine	2017	1990-2016	54	54	LT
Li et al. (20)	Clinical Transplantation	2017	Inception to 8 March 2017	6	6	LT
Koh et al. (18)	Hepatobiliary Surg Nutr	2022	Inception to 8 March 2021	35	34	LT

### Quality assessment

**Figure 2:**
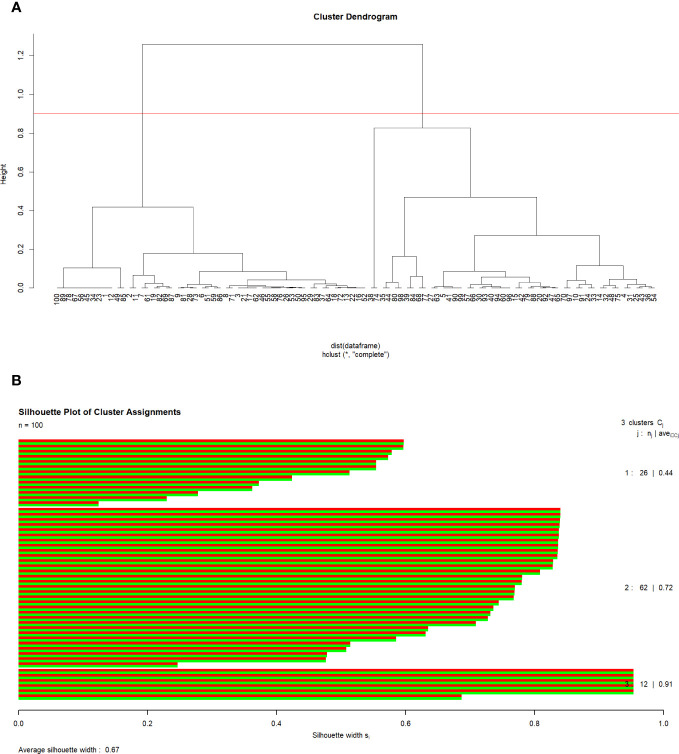
**(A)** Cluster Dendrogram. **(B)** Silhouette Plot of Cluster Assignments.

Authors of five of the eight meta-analyses cited the previously published meta-analyses, and only one study had no prior studies available to cite (**Table 2**). Every included study used Medline/PubMed as part of the literature search, and nine studies also used Embase (**Table 3**). There was variation in the utilisation of other databases, but every study (excluding two) used at least two electronic databases. According to the AMSTAR quality assessment, four studies rated low quality and six critically low quality (**Table 4**). The median Pearson correlation coefficient was 0.59 (IQR 0.41-0.65) for all the included studies (**Figure 2A**). Hierarchical clustering of the r identified 3 clusters after silhouette analysis (Cluster Sizes and Average Silhouette Widths: Cluster 1 (26 data points): Average Silhouette Width of 0.443; Cluster 2 (62 data points): Average Silhouette Width of 0.724; Cluster 3 (12 data points): Average Silhouette Width of 0.909); (Median: 0.7341 IQR: 0.5543- 0.8369; Mean: 0.6731 Range 0.1244-0.9534. (**Figure 2B**).

**Table 2:** Number of meta-analyses.

Authors	Publication Year	Date of Last Literature Search (mo/yr)	No. of Meta-Analyses Possible to Cite	No. of Meta-Analyses Cited
Hong-Yu Li (19)	2012	01/04/2010	1	0
Mashaal Dhir (17)	2012	31/03/2011	0	0
Atiq Rahman (11)	2012	01/03/2012	2	0
Zheng Zheng (25)	2014	01/04/2012	4	0
Xin-Sen Xu (24)	2014	01/07/2012	5	1
Andrea Proneth (22)	2014	01/09/2013	6	1
Benjamin Menahem (21)	2017	01/12/2016	7	0
Markus B. Schoenberg (23)	2017	01/03/2017	8	3
Wei Li (20)	2018	01/06/2017	9	1
Jin Hean Koh (18)	2022	01/03/2021	11	1

**Table 3:** Search methodology.

Authors	Year of Publication	Medline/PubMed	Embase	Cochrane Library	Other	Language Limitations
Mashaal Dhir (17)	2012	yes	no	no	no	Only English
Atiq Rahman (11)	2012	yes	yes	yes	no	no
Hong-Yu Li (19)	2012	yes	yes	yes	no	Only English
Zheng Zheng (25)	2014	yes	yes	yes	no	nr
Andrea Proneth (22)	2014	yes	yes	yes	no	Only English
Xin-Sen Xu (24)	2014	yes	yes	yes	no	Only English
Benjamin Menahem (21)	2017	yes	yes	yes	no	Only English
Markus B. Schoenberg (23)	2017	yes	yes	no	no	Only English
Wei Li (20)	2017	yes	yes	yes	no	nr
Jin Hean Koh (18)	2022	yes	yes	no	no	Only English

**Table 4:** Amstar 2 evaluation.

Domains	Items-Authors	Hong-yu (19)	Kostakis (26)	Koh (18)	Zheng Zheng (25)	Xin-sen Xu (24)	Schoenberg (23)	Proneth (22)	Rahaman (11)	Dhir (17)	Menahem (21)
	1. Did the research questions and inclusion criteria for the review include the components of PICO?	yes	yes	yes	yes	yes	yes	yes	yes	yes	yes
**Critical**	2. Did the report of the review contain an explicit statement that the review methods were established prior to the conduct of the review and did the report justify any significant deviations from the protocol?	yes	no	yes	yes	yes	yes	Partial yes	yes	yes	yes
	3. Did the review authors explain their selection of the study designs for inclusion in the review?	yes	no	no	yes	no	no	yes	no	no	no
**Critical**	4. Did the review authors use a comprehensive literature search strategy?	yes	no	Partial yes	yes	yes	yes	yes	yes	no	Partial yes
	5. Did the review authors perform study selection in duplicate?	yes	no	yes	yes	yes	yes	yes	yes	no	yes
	6. Did the review authors perform data extraction induplicate?	yes	no	yes	yes	yes	yes	yes	yes	no	yes
**Critical**	7. Did the review authors provide a list of excluded studies and justify the exclusions?	no	no	no	no	Partial yes	Partial yes	yes	Partial Yes	no	no
	8. Did the review authors describe the included studies in adequate detail?	Partial yes	Partial yes	yes	Partial yes	Partial yes	yes	Partial yes	yes	yes	yes
**Critical**	9. Did the review authors use a satisfactory technique for assessing the risk of bias (RoB) in individual studies that were included in the review?	yes	no	no	yes	no	Partial yes	yes	yes	no	no
	10. Did the review authors report on the sources of funding for the studies included in the review?	no	no	no	no	no	no	no	no	no	no
**Critical**	11. If meta-analysis was performed did the review authors use appropriate methods for statistical combination of results?	yes	yes	yes	yes	yes	yes	yes	yes	yes	yes
	12. If meta-analysis was performed, did the review authors assess the potential impact of RoB in individual studies on the results of the meta-analysis or other evidence synthesis?	yes	no	no	yes	no	yes	yes	yes	yes	no
**Critical**	13. Did the review authors account for RoB in individual studies when interpreting/discussing the results of the review?	yes	no	no	no	no	yes	yes	no	yes	no
	14. Did the review authors provide a satisfactory explanation for, and discussion of, any heterogeneity observed in the results of the review?	yes	yes	yes	no	yes	yes	no	yes	yes	no
**Critical**	15. If they performed quantitative synthesis did the review authors carry out an adequate investigation of publication bias (small study bias) and discuss its likely impact on the results of the review?	yes	no	no	yes	no	no	no	yes	yes	no
	16. Did the review authors report any potential sources of conflict of interest, including any funding they received for conducting the review?	no	no	yes	yes	yes	yes	no	no	yes	yes
	**Overall AMSTAR 2 Rating**	Low quality	Critially Low quality	Critially Low quality	Critially Low quality	Critially Low quality	Low quality	Low quality	Low quality	Critially Low quality	Critially Low quality

### Primary outcome

#### 5 years overall survival

**Figure 3:**
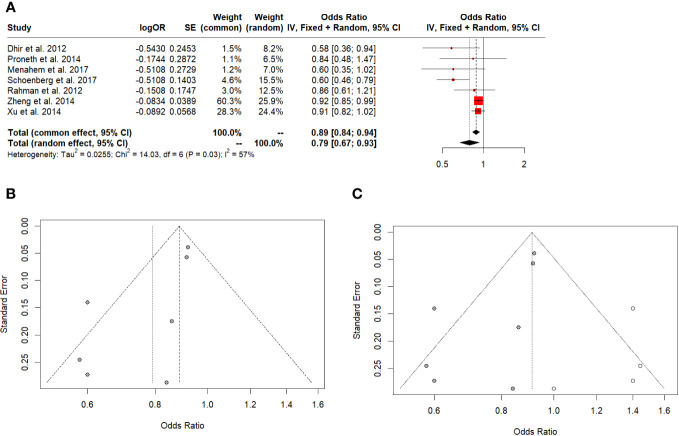
**(A)** 5-year overall survival in all HCC. **(B)** Funnel plot. **(C)** Funnel plot after Trim-and-fill.

LT showed better 5-year survival in all HCC (Odd Ratio (OR): 0.79; 95% CI: 0.67-0.93, I^2:57%), (**Figure 3A**), Egger’s test showed a significant funnel plot asymmetry (t = -2.62, df = 5, p = 0.0468). Begg’s test did not find funnel plot asymmetry (z = -1.05, p = 0.2931) (**Figure 3B**). After the 5-year survival Trim-and-fill method, both Egger’s and Begg’s tests did not show evidence of publication bias (t = -0.07, df = 9, p-value = 0.9437 and z = -0.08, p-value = 0.9372, respectively) (**Figure 3C**).

#### 5 years disease survival

**Figure 4:**
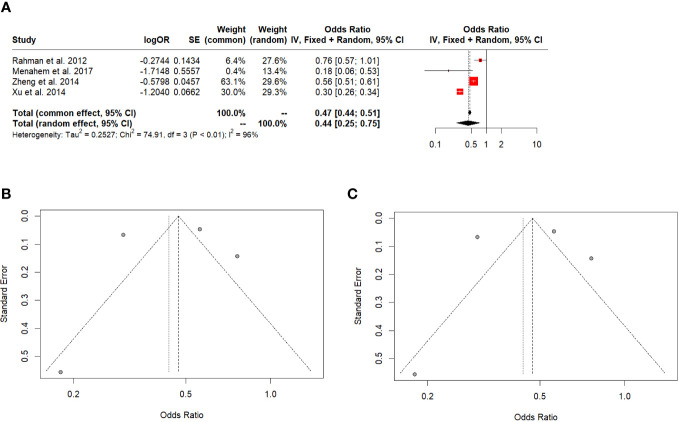
**(A)** 5-year disease-free survival in all HCC. **(B)** Funnel plot. **(C)** Funnel plot after Trim-and-fill.

DFS favoured LT for all HCC (OR: 0.44; 95% CI: 0.25-0.75, I^2:96%) (**Figure 4A**). The Egger’s test (t = 0.02, df = 3, p-value = 0.9879) and Begg’s test (z = -0.68, p-value = 0.4969) did not indicate significant publication bias in the original analysis (**Figure 4B**). After applying the Trim-and-fill method, the Egger’s test (t = 0.02, df = 3, p-value = 0.9879) and Begg’s test (z = -0.76, p-value = 0.4485) still did not show significant evidence of publication bias (**Figure 4C**).

### HR Overall and disease-free survival

**Figure 5:**
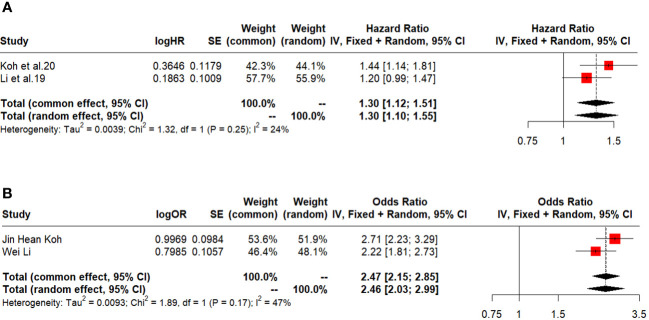
**(A)** HR overall survival. **(B)** HR disease free survival.

Two studies reported the HR for overall and disease-free survival favouring LT over liver resection (1.30, 95% CI: 1.10-1.55, I^2: 24% and 2.46, 95% CI: 2.03-2.99, I^2: 47%) (**Figures 5A, B**).

### Secondary outcomes

**Figure 6:**
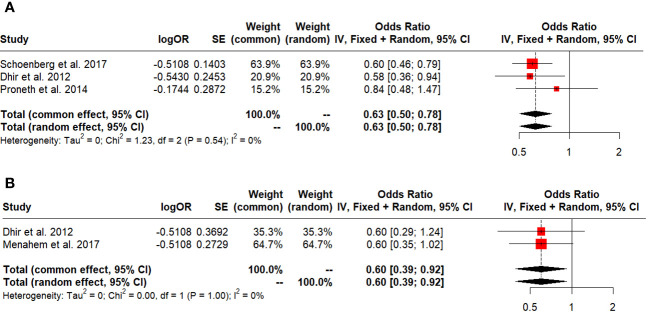
**(A)** Five-year survival in early HCC. **(B)** Five-year survival in ITT.

**Figure 7:**
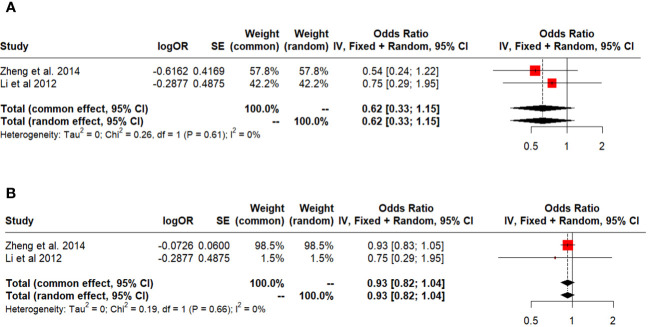
**(A)** Salvage vs primary (overall). **(B)** Salvage vs primary (disease free).

Five-year survival in early HCC and ITT was 0.63 (95% CI: 0.50-0.78, I^2:0%), (**Figure 6A**) and 0.60 (95% CI: 0.39-0.92, I^2:0%), respectively (**Figure 6B**). Salvage LT vs. Primary LT did not differ between 5-year survival and DFS (OR: 0.62; 95% CI: 0.33-1.15, I^2:0% and 0.93; 95% CI: 0.82-1.04, I^2:0%) (**Figures 7A, B**).

## Discussion

Comparing the outcomes of LT and LR in HCC is crucial because it can inform the decision-making process for selecting the most appropriate treatment option for individual patients (1, 11, 21). By identifying the best treatment between LT and LR, healthcare providers improve the patient’s overall survival and quality of life. Furthermore, there is a shortage of donor organs worldwide, so optimising organ allocation is central to HCC. In some cases, LR may be a viable alternative to LT as a definitive treatment, especially for patients with early-stage HCC and those with limited underlying liver disease or bridge therapy in case of cancer recurrences (10, 27–29). The study included a large cohort of patients, which is a relatively large sample size and may increase the reliability of the findings.

Furthermore, the study conducted a systematic review and meta-analysis of multiple meta-analyses, which may provide a more comprehensive picture of the topic. Also, the study conducted subgroup analyses for different types of HCC and liver transplantation, which may help identify specific factors that influence outcomes.

LT showed better OS and DFS than LR for HCC. However, survival after retransplantation for cancer recurrences was equal to primary LT for HCC. The finding agreed with the included meta-analyses, independently from the correlation matrix and the cluster analysis.

The results of the meta-analysis provide valuable insights into the comparative effectiveness of liver transplantation (LT) and liver resection for hepatocellular carcinoma (HCC) in terms of 5-year overall survival, disease-free survival, and hazard ratio (HR) for overall survival. These findings align with the evolving body of research in the field, which examines the optimal treatment approaches for HCC patients.

While the meta-analysis indicates funnel plot asymmetry through Egger’s test, this could suggest the presence of publication bias that may skew the results. Using the Trim-and-fill method to address publication bias enhances the reliability of the findings. The favourable disease-free survival outcomes favouring LT over liver resection for all HCC cases align with previous research suggesting that LT can lead to more extended periods without recurrence (30, 31). The absence of significant publication bias in the initial analysis and after using the Trim-and-fill method adds confidence to these findings.

Furthermore, the HR analysis suggests that LT may be associated with better overall survival than liver resection, as the HR favours LT. The I^2 value of 24% suggests moderate heterogeneity, indicating relatively consistent results among the studies included.

The quality assessment of the included studies reveals that there was at least a critical flaw in the meta-analysis methodology. Many of the studies under consideration did not adequately address the potential risks of bias in their analyses, nor did they thoroughly discuss how these biases might influence the outcomes reported in the review. This oversight raises concerns about the robustness and reliability of the findings presented in these studies (32). Biases, whether related to study design, data collection, or reporting, can introduce systematic errors that may distort the overall conclusions of a meta-analysis. Failing to acknowledge and address these biases can undermine the validity and credibility of the study’s results. It is essential for future research to comprehensively evaluate and report on the potential biases and their potential impact to ensure the accuracy and reliability of the meta-analytic findings.

There was some heterogeneity in the data, particularly in the DFS analysis, possibly due to differences in study design and patient populations. Therefore, despite the present findings, individual patient factors and clinical considerations should still be considered when determining the most appropriate treatment approach for HCC (31).

The correlation analysis of the present study indicates a moderate association level between the variables, while hierarchical clustering identified three distinct clusters based on the correlation coefficients. The integration of hierarchical clustering analysis to validate the consistency of findings adds further strength to the results. The silhouette analysis suggests these clusters are well-defined, with different data points forming cohesive groups. The three clusters showed good separation and assignment of data points to clusters, confirming a consistent agreement among the meta-analyses about the advantage of LT over LR, independently from the included studies.

Several potential sources of bias in this study should be considered. While the results and conclusions of the study may provide valuable insights into the overall management of HCC, it is essential to consider the heterogeneity of the patient population and the specific clinical contexts when interpreting the findings for different subgroups of patients. A limitation of the present study was the difficulties in drawing the same conclusions for patients with HCC within or outside Milan criteria, undergoing a first or a salvage transplantation. Similarly, whether the manuscript included three meta-analyses, reporting outcomes in ITT patients, the lack of robust data may result in a positive outcome for the LT group and in a disadvantage in the LR group. Another potential source of bias is measurement bias, as the determination of survival and disease-free survival may be affected by factors such as follow-up time, surveillance protocols, and the definition of recurrence. Finally, there may be publication bias, as studies with negative or null findings may be less likely to be published or included in systematic reviews and meta-analyses (33, 34).

By systematically analysing the citation matrix, the authors identified clusters of meta-analysis indicating potential overlap or duplication. However, the association was moderate, and the primary outcomes results consistent. The integration of hierarchical clustering analysis to validate the consistency of findings added further strength to the results. The silhouette analysis suggested these clusters were well-defined, with different data points forming cohesive groups. The three clusters showed good separation and assignment of data points to clusters, confirming a consistent agreement among the meta-analyses about the advantage of LT over LR, independently from the included studies.

Future research could explore the impact of patient-specific characteristics on treatment effectiveness, investigate new biomarkers for patient selection, develop individualised treatment algorithms, and assess novel therapies in combination with surgical interventions to improve outcomes.

In conclusion, the study’s findings consistently suggest that LT offers better 5-year and disease-free survival rates than LR for HCC. These results hold significance for clinical practice, as they provide insights into the most effective treatment approach for HCC patients. The study underscores the importance of addressing biases and limitations in meta-analyses and highlights potential areas for future research to enhance HCC treatment strategies.

## Data availability statement

The original contributions presented in the study are included in the article/**Supplementary Material**. Further inquiries can be directed to the corresponding author.

## Author contributions

AM: Conceptualization, Formal analysis, Methodology, Writing – original draft, Writing – review & editing. AB: Writing – original draft, Writing – review & editing. FC: Writing – original draft, Writing – review & editing. IW: Writing – original draft, Writing – review & editing. FF: Writing – original draft, Writing – review & editing. FA: Writing – original draft, Writing – review & editing. FG: Writing – original draft, Writing – review & editing.
