# Supplementary figures and images for: Liver transplantation vs liver resection in HCC: promoting extensive collaborative research through a survival meta-analysis of meta-analyses
Source: Front Oncol. 2024 Mar 18;14:1366607. doi: 10.3389/fonc.2024.1366607 (PMC10986178; doi:10.3389/fonc.2024.1366607)

Supplementary Figure 1

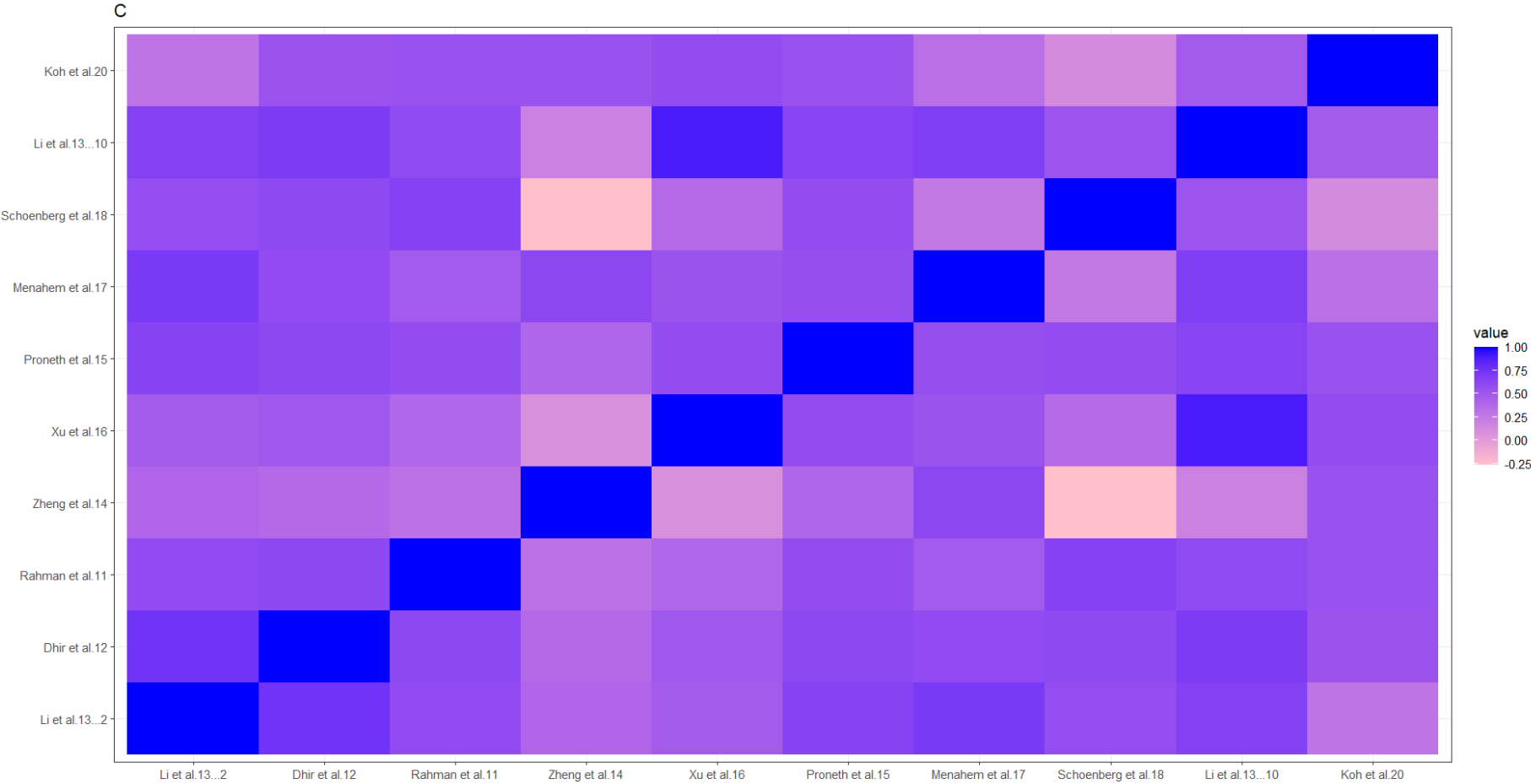

Supplement: Supplementary file 1 [file Image_1.pdf]
