# Supplementary material for: Liver transplantation vs liver resection in HCC: promoting extensive collaborative research through a survival meta-analysis of meta-analyses
Source: Front Oncol. 2024 Mar 18;14:1366607. doi: 10.3389/fonc.2024.1366607 (PMC10986178; doi:10.3389/fonc.2024.1366607)
Supplement: Supplementary file 2 [file Table_1.docx]

**Supplementary Table 1.** Citation Matrix.

| **Primary Study** | Hong-Yu Li^19^ | Mashaal Dhir^17^ | Atiq Rahman^11^ | Zheng Zheng^25^ | Xin-Sen Xu^22^ | Andrea Proneth^22^ | Benjamin Menahem^21^ | Markus B. Schoenberg^23^ | Wei Li^20^ | I.D. Kostakis^34^ | Jin Hean Koh^18^ |
| --- | --- | --- | --- | --- | --- | --- | --- | --- | --- | --- | --- |
| Adam et al. 2003 ^35^ | x |  | x | x | x |  |  | x |  |  |  |
| Adam et al. 2012 ^36^ |  |  |  |  |  |  | x | x |  |  | x |
| Aksoy et al. 2020 ^37^ |  |  |  |  |  |  |  |  |  |  | x |
| Baccaran et al. 2007 ^38^ |  | x |  | x | x |  | x | x |  |  | x |
| Belghiti et al. 2003 ^39^ | x |  |  |  |  |  |  |  |  |  |  |
| Bellavance et al. 2008 ^40^ |  | x |  | x | x |  |  | x |  |  | x |
| Bigourdan et al. 2003 ^41^ |  | x |  | x | x | x | x | x |  |  | x |
| Bismuth et al.^42^ |  |  |  |  | x |  |  |  |  |  |  |
| Borie et al. 2008 ^43^ |  |  |  |  |  | x |  |  |  |  |  |
| Bronowicki et al. 1996 ^44^ |  |  |  | x | x |  |  | x |  |  |  |
| Canter et al. 2011 ^45^ |  |  |  | x |  |  |  | x | x |  |  |
| Cha et al. 2003 ^46^ |  |  | x |  |  | x |  | x |  |  |  |
| Chan et al. 2012^47^ |  |  |  |  |  |  |  | x |  |  |  |
| Chan et al. 2013 ^48^ |  |  |  |  |  |  |  |  |  | x |  |
| Chapman et al. 2015 ^49^ |  |  |  |  |  |  |  |  |  |  | x |
| Choi et al. 2008 ^50^ |  |  |  |  |  |  |  |  |  | x |  |
| Chuan et al. 2014^51^ |  |  |  |  |  |  |  | x |  |  |  |
| Cillo et al. 2007 ^52^ |  |  |  |  | x |  |  |  |  |  |  |
| Closset et al. 1999 ^53^ |  |  |  |  |  |  |  | x |  |  |  |
| Colella et al. 1998 ^54^ |  |  |  |  |  |  |  |  |  |  |  |
| Concejero et al 2008 ^55^ | x |  |  |  |  |  |  |  |  |  |  |
| Dai et al. 2014 ^56^ |  |  |  |  |  |  |  | x |  |  | x |
| De Carlis et al. 2001 ^57^ |  |  | x |  |  |  |  |  |  |  |  |
| De Carlis et al. 2003 ^58^ |  |  |  |  | x |  |  | x |  |  |  |
| Del Gaudio et al. 2008 ^59^ | x | x | x | x | x | x |  |  |  |  |  |
| Dima et al 2009 ^60^ |  |  |  |  |  |  |  | x |  |  |  |
| El-Gazzaz et al. 2000 ^61^ |  |  |  | x |  |  |  |  |  |  |  |
| Facciuto et al. 2008 ^62^ | x |  |  | x |  | x |  | x | x |  | x |
| Fan et al. 2010 ^63^ |  |  |  | x |  |  |  | x | x |  |  |
| Fan et al. 2011 ^64^ |  |  |  | x |  |  |  |  |  |  | x |
| Farinati et al. 2001 ^65^ |  |  |  | x |  |  |  |  |  |  |  |
| Figueras et al. 2000 ^66^ |  |  | x | x | x |  |  |  |  |  |  |
| Foltys et al. 2014 ^67^ |  |  |  |  |  |  |  | x |  |  |  |
| Franssen et al 2014 ^68^ |  |  |  |  |  |  |  | x |  |  |  |
| Fuks et al. 2011 ^69^ |  |  |  | x |  |  |  |  |  |  |  |
| Graham et al., 2013 ^70^ |  |  |  |  |  |  |  |  |  |  | x |
| Harada 2012 ^71^ |  |  |  |  |  |  |  | x |  |  |  |
| Ho et al. 2012 ^72^ |  |  |  | x |  |  |  | x |  |  |  |
| Hsueh et al., 2016 ^73^ |  |  |  |  |  |  |  | x |  |  | x |
| Huang et al. 2016 ^74^ |  |  |  |  |  |  |  |  |  |  | x |
| Hwang et al., 2007 ^75^ | x |  |  |  |  |  |  |  |  |  | x |
| Iwatsuki et al. 1991 ^76^ |  |  |  |  | x |  |  | x |  |  |  |
| Jiang et al., 2014 ^77^ |  |  |  |  |  |  | x | x |  |  | x |
| Kaido et al. 2012 ^78^ |  |  |  |  |  |  |  | x |  |  |  |
| Kim et al. 2008 ^79^ | x |  |  |  |  |  |  |  |  |  |  |
| Koniaris et al. 2011 ^80^ |  |  | x | x | x | x | x | x |  |  | x |
| Kooby et al. 2008 ^81^ |  |  |  | x |  |  |  | x |  |  |  |
| Krenzien et al., 2018 ^28^ |  |  |  |  |  |  |  |  |  |  | x |
| Kuroda et al.v2011 ^82^ |  |  |  |  |  |  |  |  |  |  | x |
| Langer et al. 1994 ^83^ |  |  |  | x |  |  |  | x |  |  |  |
| Launois et al. 1996 ^84^ |  |  |  | x |  |  |  |  |  |  |  |
| Lee et al. 2010 ^27^ |  | x | x | x | x |  |  |  | x |  | x |
| Lei et al 2013 ^85^ |  |  |  |  |  |  |  | x |  |  |  |
| Li et al. 2014 ^86^ |  |  |  |  |  |  |  | x |  |  | x |
| Li et al. 2017 ^87^ |  |  |  |  |  |  |  |  |  |  | x |
| Lim et al 2017 ^88^ |  |  |  |  |  |  |  |  |  | x |  |
| Llovet et al. 1999 ^89^ |  | x |  | x | x | x | x | x |  |  | x |
| Margarit et al. 2005 ^90^ | x | x | x | x | x |  |  | x |  |  |  |
| Mazziotti et al. 1998 ^91^ |  |  |  | x |  |  |  | x |  |  |  |
| Meyerovich et al. 2019 ^92^ |  |  |  |  |  |  |  |  |  |  | x |
| Michel et al. 1997 ^93^ |  |  |  | x | x |  |  | x |  |  |  |
| Michelakos et al. 2019 ^94^ |  |  |  |  |  |  |  |  |  |  | x |
| Moon et al. 2007 ^95^ |  |  |  | x |  |  |  | x |  |  | x |
| Ng et al. 2008 ^96^ |  |  |  |  |  |  |  |  |  | x |  |
| Obed et al. 2008 ^97^ |  |  |  | x |  |  |  | x |  |  |  |
| Otto et al. 1998 ^98^ |  |  |  |  | x |  |  | x |  |  |  |
| Park et al., 2017 ^99^ |  |  |  |  |  |  |  |  |  |  | x |
| Perry et al 2007 ^100^ |  |  |  |  |  |  |  | x |  |  |  |
| Peters et al. 2017 ^101^ |  |  |  |  |  |  |  |  |  |  | x |
| Philosophe et al. 1998 ^102^ |  |  |  | x | x |  |  | x |  |  |  |
| Poon et al. 2007 ^103^ |  | x | x | x | x | x |  | x |  |  |  |
| Rayya et al. 2008 ^104^ |  |  |  |  |  |  |  | x |  |  |  |
| Ringe et al. 1991 ^105^ |  |  |  | x | x |  |  | x |  |  |  |
| Ruzzenente et al. 2009 ^106^ |  |  |  | x |  |  |  |  |  |  |  |
| Sangro et al. 1998 ^107^ |  |  |  | x |  |  |  | x |  |  |  |
| Sapisochin et al. 2013 ^108^ |  |  |  |  |  |  | x | x |  |  | x |
| Sapisochin et al., 2010 ^109^ | x |  |  |  |  |  |  |  |  |  |  |
| Scatton et al. 2008 ^110^ |  |  |  |  |  |  | x |  |  |  |  |
| Seshadri et 2014 ^111^ |  |  |  |  |  |  |  | x |  |  |  |
| Shabahang et al. 2002 ^112^ |  |  |  |  | x |  |  | x |  |  |  |
| Shah et al. 2007 ^113^ |  | x | x | x | x | x | x | x |  |  | x |
| Shao et al. 2008 ^114^ | x |  |  |  |  |  |  |  |  |  |  |
| Shen et al. 2002 ^115^ |  |  |  |  |  |  |  |  |  | x |  |
| Shen et al. 2017 ^116^ |  |  |  |  |  |  |  |  | x |  |  |
| Sogawa et al. 2013 ^117^ |  |  |  |  |  |  | x | x |  |  | x |
| Sotiropoulos et al. 2009 ^118^ |  |  |  |  | x |  |  |  |  |  |  |
| Squires et al 2014 ^119^ |  |  |  |  |  |  |  | x |  |  |  |
| Sung et al. 2017 ^120^ |  |  |  |  |  |  |  |  |  |  | x |
| Tan et al. 1995 ^121^ |  |  |  |  | x |  |  | x |  |  |  |
| Tiao et al. 2005 ^122^ |  |  |  | x |  |  |  |  |  |  |  |
| Vargas et al. 1995 ^123^ |  |  |  |  |  |  |  | x |  |  |  |
| Vennarecci et al. 2007 ^124^ | x |  |  |  |  |  |  |  |  |  |  |
| Weimann et al. 1999 ^125^ |  | x |  | x |  |  |  | x |  |  |  |
| Wu et al. 2020 ^126^ |  |  |  |  |  |  |  |  |  |  | x |
| Yamamoto et al. 1999^127^ | x | x | x | x | x |  |  |  |  |  |  |
| Yamashita et al. 2015 ^128^ |  |  |  |  |  |  |  |  |  | x |  |
| Yang et al. 2017 ^129^ |  |  |  |  |  |  |  |  |  |  | x |
| Yokoi et al. 2006 ^130^ |  |  |  |  |  |  |  |  |  |  | x |
| Zaydfudim et al. 2016 ^131^ |  |  |  |  |  |  |  |  | x |  |  |
| Zhou et al. 2010 ^132^ |  |  |  |  | x |  |  |  |  |  | x |

**References**

11. Rahman A, Assifi MM, Pedroso FE, Maley WR, Sola JE, Lavu H, et al. Is resection equivalent to transplantation for early cirrhotic patients with hepatocellular carcinoma? A meta-analysis. J Gastrointest Surg. 2012 Oct;16(10):1897-909. doi: 10.1007/s11605-012-1973-8. Epub 2012 Jul 27. PMID: 22836922.

17. Dhir M, Lyden ER, Smith LM, Are C. Comparison of outcomes of transplantation and resection in patients with early hepatocellular carcinoma: a meta-analysis. *HPB (Oxford).* 2012;14(9): 635-645.

18. Koh, J. H., Tan, D. J. H., Ong, Y., Lim, W. H., Ng, C. H., Tay, P. W. L., et al. Liver resection versus liver transplantation for hepatocellular carcinoma within Milan criteria: a meta-analysis of 18,421 patients. *Hepatobiliary surgery and nutrition*, *11*(1), 78–93. https://doi.org/10.21037/hbsn-21-350

19. Li HY, Wei YG, Yan LN, Li B. Salvage liver transplantation in the treatment of hepatocellular carcinoma: a meta-analysis. *World J Gastroenterol.* 2012;18(19): 2415-2422.

20. Li W, Li L, Han J, Wu H. Liver transplantation vs liver resection in patients with HBV-related hepatocellular carcinoma beyond Milan criterion: A meta-analysis. *Clinical transplantation.* 2018;32(3): e13193.

21. Menahem, B., Lubrano, J., Duvoux, C., Mulliri, A., Alves, A., Costentin, C., et al. Liver transplantation versus liver resection for hepatocellular carcinoma in intention to treat: An attempt to perform an ideal meta-analysis. *Liver transplantation : official publication of the American Association for the Study of Liver Diseases and the International Liver Transplantation Society*, *23*(6), 836–844. https://doi.org/10.1002/lt.24758

22. Proneth A, Zeman F, Schlitt HJ, Schnitzbauer AA. Is resection or transplantation the ideal treatment in patients with hepatocellular carcinoma in cirrhosis if both are possible? A systematic review and metaanalysis. *Ann Surg Oncol.* 2014;21(9): 3096-3107.

23. Schoenberg, M. B., Bucher, J. N., Vater, A., Bazhin, A. V., Hao, J., Guba, M. O., et al. Resection or Transplant in Early Hepatocellular Carcinoma. *Deutsches Arzteblatt international*, *114*(31-32), 519–526. https://doi.org/10.3238/arztebl.2017.0519

25. Zheng, Z., Liang, W., Milgrom, D. P., Zheng, Z., Schroder, P. M., Kong, N. S., et al. Liver transplantation versus liver resection in the treatment of hepatocellular carcinoma: a meta-analysis of observational studies. Transplantation, 97(2), 227–234. <https://doi.org/10.1097/TP.0b013e3182a89383>.

27. Lee KK, Kim DG, Moon IS, Lee MD, Park JH. Liver transplantation versus liver resection for the treatment of hepatocellular carcinoma. *J Surg Oncol.* 2010;101(1): 47-53.

34. Kostakis ID, Machairas N, Prodromidou A, et al. Comparison Between Salvage Liver Transplantation and Repeat Liver Resection for Recurrent Hepatocellular Carcinoma: A Systematic Review and Meta-analysis. *Transplantation proceedings.* 2019;51(2): 433-436.

35. Adam, R., Azoulay, D., Castaing, D., Eshkenazy, R., Pascal, G., Hashizume, K., et al (2003). Liver resection as a bridge to transplantation for hepatocellular carcinoma on cirrhosis: a reasonable strategy?. *Annals of surgery*, *238*(4), 508–519. https://doi.org/10.1097/01.sla.0000090449.87109.44

36. Adam, R., Bhangui, P., Vibert, E., Azoulay, D., Pelletier, G., Duclos-Vallée, J. C., et al. (2012). Resection or transplantation for early hepatocellular carcinoma in a cirrhotic liver: does size define the best oncological strategy?. Annals of surgery, 256(6), 883–891. https://doi.org/10.1097/SLA.0b013e318273bad0

37. Aksoy, S. O., Unek, T., Sevinc, A. İ., Arslan, B., Sirin, H., Derici, Z. S., et al. (2020). Comparison of Resection and Liver Transplant in Treatment of Hepatocellular Carcinoma. *Experimental and clinical transplantation : official journal of the Middle East Society for Organ Transplantation*, *18*(6), 712–718. <https://doi.org/10.6002/ect.2017.0303>.

38. Baccarani, U., Isola, M., Adani, G. L., Benzoni, E., Avellini, C., Lorenzin, D., et al. (2008). Superiority of transplantation versus resection for the treatment of small hepatocellular carcinoma. *Transplant international : official journal of the European Society for Organ Transplantation*, *21*(3), 247–254. https://doi.org/10.1111/j.1432-2277.2007.00597.x

39. Belghiti J, Cortes A, Abdalla EK, et al. Resection prior to liver transplantation for hepatocellular carcinoma. *Ann Surg.* 2003;238(6): 885-892; discussion 892-883.

40. Bellavance, E. C., Lumpkins, K. M., Mentha, G., Marques, H. P., Capussotti, L., Pulitano, C., et al. (2008). Surgical management of early-stage hepatocellular carcinoma: resection or transplantation?. *Journal of gastrointestinal surgery : official journal of the Society for Surgery of the Alimentary Tract*, *12*(10), 1699–1708. https://doi.org/10.1007/s11605-008-0652-2

41. Bigourdan, J. M., Jaeck, D., Meyer, N., Meyer, C., Oussoultzoglou, E., Bachellier, P., et al. (2003). Small hepatocellular carcinoma in Child A cirrhotic patients: hepatic resection versus transplantation. *Liver transplantation : official publication of the American Association for the Study of Liver Diseases and the International Liver Transplantation Society*, *9*(5), 513–520. https://doi.org/10.1053/jlts.2003.50070

43. Borie, F., Bouvier, A. M., Herrero, A., Faivre, J., Launoy, G., Delafosse, P., et al. (2008). Treatment and prognosis of hepatocellular carcinoma: a population based study in France. *Journal of surgical oncology*, *98*(7), 505–509. https://doi.org/10.1002/jso.21159

44. Bronowicki, J. P., Boudjema, K., Chone, L., Nisand, G., Bazin, C., Pflumio, F., et al. (1996). Comparison of resection, liver transplantation and transcatheter oily chemoembolization in the treatment of hepatocellular carcinoma. *Journal of hepatology*, *24*(3), 293–300. https://doi.org/10.1016/s0168-8278(96)80007-9

45. Canter, R. J., Patel, S. A., Kennedy, T., D'Angelica, M. I., Jarnagin, W. R., Fong, Y., et al. (2011). Comparative analysis of outcome in patients with hepatocellular carcinoma exceeding the milan criteria treated with liver transplantation versus partial hepatectomy. *American journal of clinical oncology*, *34*(5), 466–471. https://doi.org/10.1097/COC.0b013e3181ec63dd

46. Cha, C. H., Ruo, L., Fong, Y., Jarnagin, W. R., Shia, J., Blumgart, L. H., et al. (2003). Resection of hepatocellular carcinoma in patients otherwise eligible for transplantation. *Annals of surgery*, *238*(3), 315–323. https://doi.org/10.1097/01.sla.0000086548.84705.ef

47. Chan, S. C., Fan, S. T., Chok, K. S., Cheung, T. T., Chan, A. C., Fung, J. Y., et al. (2011). Survival advantage of primary liver transplantation for hepatocellular carcinoma within the up-to-7 criteria with microvascular invasion. *Hepatology international*, *6*(3), 646–656. https://doi.org/10.1007/s12072-011-9318-3

48. Chan, A. C., Chan, S. C., Chok, K. S., Cheung, T. T., Chiu, D. W., Poon, R. T., et al. (2013). Treatment strategy for recurrent hepatocellular carcinoma: salvage transplantation, repeated resection, or radiofrequency ablation?. *Liver transplantation : official publication of the American Association for the Study of Liver Diseases and the International Liver Transplantation Society*, *19*(4), 411–419. https://doi.org/10.1002/lt.23605

49. Choi, G. H., Kim, D. H., Kang, C. M., Kim, K. S., Choi, J. S., Lee, W. J., et al. (2008). Prognostic factors and optimal treatment strategy for intrahepatic nodular recurrence after curative resection of hepatocellular carcinoma. *Annals of surgical oncology*, *15*(2), 618–629. https://doi.org/10.1245/s10434-007-9671-6

50. Choi GH, Kim DH, Kang CM, et al. Prognostic factors and optimal treatment strategy for intrahepatic nodular recurrence after curative resection of hepatocellular carcinoma. *Ann Surg Oncol.* 2008;15(2): 618-629.

51. Chuan, W., Li, C., Wen, T. F., Yan, L. N., Li, B., Liang, G. L., & Li, K. W. (2014). Short-term and long-term outcomes of surgical treatment for HCC within Milan criteria with cirrhotic portal hypertension. *Hepato-gastroenterology*, *61*(136), 2185–2190.

52. Cillo, U., Vitale, A., Brolese, A., Zanus, G., Neri, D., Valmasoni, M., et al. (2007). Partial hepatectomy as first-line treatment for patients with hepatocellular carcinoma. *Journal of surgical oncology*, *95*(3), 213–220. https://doi.org/10.1002/jso.20641

53. Closset J, Van de Stadt J, Delhaye M, El Nakadi I, Lambilliotte JP, Gelin M. Hepatocellular carcinoma: surgical treatment and prognostic variables in 56 patients. *Hepatogastroenterology.* 1999;46(29): 2914-2918.

54. Colella, G., Bottelli, R., De Carlis, L., Sansalone, C. V., Rondinara, G. F., Alberti, A., et al. (1998). Hepatocellular carcinoma: comparison between liver transplantation, resective surgery, ethanol injection, and chemoembolization. *Transplant international : official journal of the European Society for Organ Transplantation*, *11 Suppl 1*, S193–S196. https://doi.org/10.1007/s001470050459

55. Dai, Y., Li, C., Wen, T. F., & Yan, L. N. (2014). Comparison of liver resection and transplantation for Child-pugh A cirrhotic patient with very early hepatocellular carcinoma and portal hypertension. *Pakistan journal of medical sciences*, *30*(5), 996–1000. https://doi.org/10.12669/pjms.305.5038

57. De Carlis L, Sammartino C, Giacomoni A. [Surgical treatment of hepatocellular carcinoma: resection or transplantation? Results of a multivariate analysis]. *Chirurgia italiana.* 2001;53(5): 579-586.

58. De Carlis, L., Giacomoni, A., Pirotta, V., Lauterio, A., Slim, A. O., Sammartino, et al. (2003). Surgical treatment of hepatocellular cancer in the era of hepatic transplantation. *Journal of the American College of Surgeons*, *196*(6), 887–897. https://doi.org/10.1016/S1072-7515(03)00140-6

59. Del Gaudio, M., Ercolani, G., Ravaioli, M., Cescon, M., Lauro, A., Vivarelli, M., et al. (2008). Liver transplantation for recurrent hepatocellular carcinoma on cirrhosis after liver resection: University of Bologna experience. *American journal of transplantation : official journal of the American Society of Transplantation and the American Society of Transplant Surgeons*, *8*(6), 1177–1185. https://doi.org/10.1111/j.1600-6143.2008.02229.x

60. Dima, S. O., Iacob, S., Botea, F., Matei, E., Dorobanfu, B., Vasile, S., et al. (2009). Multimodal treatment of hepatocellular carcinoma: an eastern European experience. *Hepato-gastroenterology*, *56*(96), 1696–1703.

61. El-Gazzaz, G., Wong, W., El-Hadary, M. K., Gunson, B. K., Mirza, D. F., et al. (2000). Outcome of liver resection and transplantation for fibrolamellar hepatocellular carcinoma. *Transplant international : official journal of the European Society for Organ Transplantation*, *13 Suppl 1*, S406–S409. https://doi.org/10.1007/s001470050372

62. Facciuto, M. E., Koneru, B., Rocca, J. P., Wolf, D. C., Kim-Schluger, L., Visintainer, P., et al. (2008). Surgical treatment of hepatocellular carcinoma beyond Milan criteria. Results of liver resection, salvage transplantation, and primary liver transplantation. *Annals of surgical oncology*, *15*(5), 1383–1391. https://doi.org/10.1245/s10434-008-9851-z

63. Fan, H. L., Chen, T. W., Hsieh, C. B., Jan, H. C., His, S. C., De-Chuan, C., et al. (2010). Liver transplantation is an alternative treatment of hepatocellular carcinoma beyond the Milan criteria. *American journal of surgery*, *200*(2), 252–257. https://doi.org/10.1016/j.amjsurg.2009.07.049

64. Fan, S. T., Poon, R. T., Yeung, C., Lam, C. M., Lo, C. M., Yuen, W. K., et al. (2011). Outcome after partial hepatectomy for hepatocellular cancer within the Milan criteria. *The British journal of surgery*, *98*(9), 1292–1300. https://doi.org/10.1002/bjs.7583

65. Farinati F, Gianni S, Marin G, Fagiuoli S, Rinaldi M, Naccarato R. Does the choice of treatment influence survival of patients with small hepatocellular carcinoma in compensated cirrhosis? *Eur J Gastroenterol Hepatol.* 2001;13(10): 1217-1224.

66. Figueras, J., Jaurrieta, E., Valls, C., Ramos, E., Serrano, T., Rafecas, et al. (2000). Resection or transplantation for hepatocellular carcinoma in cirrhotic patients: outcomes based on indicated treatment strategy. *Journal of the American College of Surgeons*, *190*(5), 580–587. https://doi.org/10.1016/s1072-7515(00)00251-9

67. Foltys, D., Zimmermann, T., Kaths, M., Strempel, M., Heise, M., Hoppe-Lotichius, M., et al. (2014). Hepatocellular carcinoma in Child's A cirrhosis: a retrospective analysis of matched pairs following liver transplantation vs. liver resection according to the intention-to-treat principle. *Clinical transplantation*, *28*(1), 37–46. https://doi.org/10.1111/ctr.12273

68. Franssen, B., Alshebeeb, K., Tabrizian, P., Marti, J., Pierobon, E. S., Lubezky, N., et al. (2014). Differences in surgical outcomes between hepatitis B- and hepatitis C-related hepatocellular carcinoma: a retrospective analysis of a single North American center. *Annals of surgery*, *260*(4), 650–658. https://doi.org/10.1097/SLA.0000000000000917

69. Fuks D, Dokmak S, Paradis V, Diouf M, Durand F, Belghiti J. Benefit of initial resection of hepatocellular carcinoma followed by transplantation in case of recurrence: an intention-to-treat analysis. *Hepatology.* 2012;55(1): 132-140.

70. Graham JA, Newman DA, Smirniotopolous J, Shetty K, Slidell MB, Johnson LB. Transplantation for hepatocellular carcinoma in younger patients has an equivocal survival advantage as compared with resection. *Transplantation proceedings.* 2013;45(1): 265-271.

71. Harada N, Shirabe K, Ikeda Y, Korenaga D, Takenaka K, Maehara Y. Surgical management of hepatocellular carcinoma in Child-Pugh class B cirrhotic patients: hepatic resection and/or microwave coagulation therapy versus living donor liver transplantation. *Annals of transplantation.* 2012;17(4): 11-20.

72. Ho CM, Lee PH, Chen CL, Ho MC, Wu YM, Hu RH. Long-term outcomes after resection versus transplantation for hepatocellular carcinoma within UCSF criteria. *Ann Surg Oncol.* 2012;19(3): 826-833.

73. Hsueh, K. C., Lee, T. Y., Kor, C. T., Chen, T. M., Chang, T. M., Yang, S. F., et al. (2016). The role of liver transplantation or resection for patients with early hepatocellular carcinoma. *Tumour biology : the journal of the International Society for Oncodevelopmental Biology and Medicine*, *37*(3), 4193–4201. https://doi.org/10.1007/s13277-015-4243-z

74. Huang, Z. Y., Liang, B. Y., Xiong, M., Dong, K. S., Zhang, Z. Y., Zhang, E. L., et al. (2016). Severity of cirrhosis should determine the operative modality for patients with early hepatocellular carcinoma and compensated liver function. *Surgery*, *159*(2), 621–631. https://doi.org/10.1016/j.surg.2015.09.002

75. Hwang, S., Lee, S. G., Moon, D. B., Ahn, C. S., Kim, K. H., Lee, Y. J., et al. (2007). Salvage living donor liver transplantation after prior liver resection for hepatocellular carcinoma. *Liver transplantation : official publication of the American Association for the Study of Liver Diseases and the International Liver Transplantation Society*, *13*(5), 741–746. https://doi.org/10.1002/lt.21157

76. Iwatsuki, S., Starzl, T. E., Sheahan, D. G., Yokoyama, I., Demetris, A. J., Todo, S., et al. (1991). Hepatic resection versus transplantation for hepatocellular carcinoma. *Annals of surgery*, *214*(3), 221–229. https://doi.org/10.1097/00000658-199109000-00005

77. Jiang L, Liao A, Wen T, Yan L, Li B, Yang J. Living donor liver transplantation or resection for Child-Pugh A hepatocellular carcinoma patients with multiple nodules meeting the Milan criteria. *Transplant international : official journal of the European Society for Organ Transplantation.* 2014;27(6): 562-569.

78. Kaido, T., Morita, S., Tanaka, S., Ogawa, K., Mori, A., Hatano, E., et al. (2015). Long-term outcomes of hepatic resection versus living donor liver transplantation for hepatocellular carcinoma: a propensity score-matching study. *Disease markers*, *2015*, 425926. https://doi.org/10.1155/2015/425926

79. Kim BW, Park YK, Kim YB, Wang HJ, Kim MW. Salvage liver transplantation for recurrent hepatocellular carcinoma after liver resection: feasibility of the Milan criteria and operative risk. *Transplantation proceedings.* 2008;40(10): 3558-3561.

80. Koniaris, L. G., Levi, D. M., Pedroso, F. E., Franceschi, D., Tzakis, A. G., Santamaria-Barria, J. A., et al. (2011). Is surgical resection superior to transplantation in the treatment of hepatocellular carcinoma?. *Annals of surgery*, *254*(3), 527–538. https://doi.org/10.1097/SLA.0b013e31822ca66f

81. Kooby, D. A., Egnatashvili, V., Graiser, M., Delman, K. A., Kauh, J., Wood, W. C., et al. (2008). Changing management and outcome of hepatocellular carcinoma: evaluation of 501 patients treated at a single comprehensive center. *Journal of surgical oncology*, *98*(2), 81–88. https://doi.org/10.1002/jso.21049

82. Kuroda S, Tashiro H, Kobayashi T, Oshita A, Amano H, Ohdan H. Selection criteria for hepatectomy in patients with hepatocellular carcinoma classified as Child-Pugh class B. *World journal of surgery.* 2011;35(4): 834-841.

83. Langer B, Greig PD, Taylor BR. Surgical resection and transplantation for hepatocellular carcinoma. *Cancer treatment and research.* 1994;69: 231-240.

84. Launois B, Chauvin J, Machado ML, Bourdonnec P, Campion JP, Bardaxoglou E. [Surgical treatment of hepatocarcinoma in cirrhosis]. *Annales de gastroenterologie et d'hepatologie.* 1996;32(1): 35-39; discussion 39-40.

85. Lei JY, Yan LN, Wang WT. Transplantation vs resection for hepatocellular carcinoma with compensated liver function after downstaging therapy. *World J Gastroenterol.* 2013;19(27): 4400-4408.

86. Li, C., Zhu, W. J., Wen, T. F., Dai, Y., Yan, L. N., Li, B., et al. (2014). Child-Pugh A hepatitis B-related cirrhotic patients with a single hepatocellular carcinoma up to 5 cm: liver transplantation vs. resection. *Journal of gastrointestinal surgery : official journal of the Society for Surgery of the Alimentary Tract*, *18*(8), 1469–1476. https://doi.org/10.1007/s11605-014-2550-0

87. Li, C., Liu, J. Y., Peng, W., Wen, T. F., Yan, L. N., Yang, J. Y., et al. (2017). Liver resection *versus* transplantation for multiple hepatocellular carcinoma: a propensity score analysis. *Oncotarget*, *8*(46), 81492–81500. https://doi.org/10.18632/oncotarget.20623

88. Lim, C., Shinkawa, H., Hasegawa, K., Bhangui, P., Salloum, C., Gomez Gavara, C., et al. (2017). Salvage liver transplantation or repeat hepatectomy for recurrent hepatocellular carcinoma: An intent-to-treat analysis. *Liver transplantation : official publication of the American Association for the Study of Liver Diseases and the International Liver Transplantation Society*, *23*(12), 1553–1563. https://doi.org/10.1002/lt.24952

89. Llovet JM, Fuster J, Bruix J. Intention-to-treat analysis of surgical treatment for early hepatocellular carcinoma: resection versus transplantation. *Hepatology.* 1999;30(6): 1434-1440.

90. Margarit C, Escartin A, Castells L, Vargas V, Allende E, Bilbao I. Resection for hepatocellular carcinoma is a good option in Child-Turcotte-Pugh class A patients with cirrhosis who are eligible for liver transplantation. *Liver transplantation : official publication of the American Association for the Study of Liver Diseases and the International Liver Transplantation Society.* 2005;11(10): 1242-1251.

91. Mazziotti A, Grazi GL, Cavallari A. Surgical treatment of hepatocellular carcinoma on cirrhosis: a Western experience. *Hepatogastroenterology.* 1998;45 Suppl 3: 1281-1287.

92. Meyerovich, G., Goykhman, Y., Nakache, R., Nachmany, I., Lahat, G., Shibolet, O., et al. (2019). Resection vs Transplant Listing for Hepatocellular Carcinoma: An Intention-to-Treat Analysis. *Transplantation proceedings*, *51*(6), 1867–1873. https://doi.org/10.1016/j.transproceed.2019.02.030

93. Michel, J., Suc, B., Montpeyroux, F., Hachemanne, S., Blanc, P., Domergue, J., et al. (1997). Liver resection or transplantation for hepatocellular carcinoma? Retrospective analysis of 215 patients with cirrhosis. *Journal of hepatology*, *26*(6), 1274–1280. https://doi.org/10.1016/s0168-8278(97)80462-x

94. Michelakos, T., Xourafas, D., Qadan, M., Pieretti-Vanmarcke, R., Cai, L., Patel, M. S., et al. (2019). Hepatocellular Carcinoma in Transplantable Child-Pugh A Cirrhotics: Should Cost Affect Resection vs Transplantation?. *Journal of gastrointestinal surgery : official journal of the Society for Surgery of the Alimentary Tract*, *23*(6), 1135–1142. https://doi.org/10.1007/s11605-018-3946-z

95. Moon DB, Lee SG, Hwang S. Liver transplantation for hepatocellular carcinoma: single nodule with Child-Pugh class A sized less than 3 cm. *Dig Dis.* 2007;25(4): 320-328.

96. Ng KK, Lo CM, Liu CL, Poon RT, Chan SC, Fan ST. Survival analysis of patients with transplantable recurrent hepatocellular carcinoma: implications for salvage liver transplant. *Arch Surg.* 2008;143(1): 68-74; discussion 74.

97. Obed, A., Tsui, T. Y., Schnitzbauer, A. A., Obed, M., Schlitt, H. J., Becker, H., et al. (2008). Liver transplantation as curative approach for advanced hepatocellular carcinoma: is it justified?. *Langenbeck's archives of surgery*, *393*(2), 141–147. https://doi.org/10.1007/s00423-007-0250-x

98. Otto G, Heuschen U, Hofmann WJ, Krumm G, Hinz U, Herfarth C. Survival and recurrence after liver transplantation versus liver resection for hepatocellular carcinoma: a retrospective analysis. *Ann Surg.* 1998;227(3): 424-432.

99. Park, M. S., Lee, K. W., Kim, H., Choi, Y. R., Hong, G., Yi, N. J., et al. (2017). Primary Living-donor Liver Transplantation Is Not the Optimal Treatment Choice in Patients With Early Hepatocellular Carcinoma With Poor Tumor Biology. *Transplantation proceedings*, *49*(5), 1103–1108. https://doi.org/10.1016/j.transproceed.2017.03.016

100. Perry, J. F., Charlton, B., Koorey, D. J., Waugh, R. C., Gallagher, P. J., Crawford, M. D., et al. (2007). Outcome of patients with hepatocellular carcinoma referred to a tertiary centre with availability of multiple treatment options including cadaveric liver transplantation. *Liver international : official journal of the International Association for the Study of the Liver*, *27*(9), 1240–1248. https://doi.org/10.1111/j.1478-3231.2007.01569.x

101. Peters NA, Javed AA, He J, Wolfgang CL, Weiss MJ. Association of socioeconomics, surgical therapy, and survival of early stage hepatocellular carcinoma. *The Journal of surgical research.* 2017;210: 253-260.

106. Ruzzenente, A., Capra, F., Pachera, S., Iacono, C., Piccirillo, G., Lunardi, M., Pet al. (2009). Is liver resection justified in advanced hepatocellular carcinoma? Results of an observational study in 464 patients. *Journal of gastrointestinal surgery : official journal of the Society for Surgery of the Alimentary Tract*, *13*(7), 1313–1320. https://doi.org/10.1007/s11605-009-0903-x

107. Sangro, B., Herráiz, M., Martínez-González, M. A., Bilbao, I., Herrero, I., Beloqui, O., et al. (1998). Prognosis of hepatocellular carcinoma in relation to treatment: a multivariate analysis of 178 patients from a single European institution. *Surgery*, *124*(3), 575–583.

108. Sapisochin, G., Castells, L., Dopazo, C., Bilbao, I., Minguez, B., Lázaro, J. L., et al. (2013). Single HCC in cirrhotic patients: liver resection or liver transplantation? Long-term outcome according to an intention-to-treat basis. *Annals of surgical oncology*, *20*(4), 1194–1202. https://doi.org/10.1245/s10434-012-2655-1

109. Sapisochin, G., Bilbao, I., Balsells, J., Dopazo, C., Caralt, M., Lázaro, J. L., et al. (2010). Optimization of liver transplantation as a treatment of intrahepatic hepatocellular carcinoma recurrence after partial liver resection: experience of a single European series. *World journal of surgery*, *34*(9), 2146–2154. https://doi.org/10.1007/s00268-010-0583-4

110. Scatton, O., Zalinski, S., Terris, B., Lefevre, J. H., Casali, A., Massault, P. P., et al. (2008). Hepatocellular carcinoma developed on compensated cirrhosis: resection as a selection tool for liver transplantation. *Liver transplantation : official publication of the American Association for the Study of Liver Diseases and the International Liver Transplantation Society*, *14*(6), 779–788. https://doi.org/10.1002/lt.21431

111. Seshadri, R. M., Besur, S., Niemeyer, D. J., Templin, M., McKillop, I. H., Swan, R. Z., et al. (2014). Survival analysis of patients with stage I and II hepatocellular carcinoma after a liver transplantation or liver resection. *HPB : the official journal of the International Hepato Pancreato Biliary Association*, *16*(12), 1102–1109. https://doi.org/10.1111/hpb.12300

112. Shabahang, M., Franceschi, D., Yamashiki, N., Reddy, R., Pappas, P. A., Aviles, K., et al. (2002). Comparison of hepatic resection and hepatic transplantation in the treatment of hepatocellular carcinoma among cirrhotic patients. *Annals of surgical oncology*, *9*(9), 881–886. https://doi.org/10.1007/BF02557525

113. Shah, S. A., Cleary, S. P., Tan, J. C., Wei, A. C., Gallinger, S., Grant, D. R., et al. (2007). An analysis of resection vs transplantation for early hepatocellular carcinoma: defining the optimal therapy at a single institution. *Annals of surgical oncology*, *14*(9), 2608–2614. https://doi.org/10.1245/s10434-007-9443-3

114. Shao Z, Lopez R, Shen B, Yang GS. Orthotopic liver transplantation as a rescue operation for recurrent hepatocellular carcinoma after partial hepatectomy. *World J Gastroenterol.* 2008;14(27): 4370-4376.

115. Shen BY, Li HW, Regimbeau JM, Belghiti J. Recurrence after resection of hepatocellular carcinoma. *Hepatobiliary & pancreatic diseases international : HBPD INT.* 2002;1(3): 401-405.

116. Shen, J. Y., Li, C., Wen, T. F., Yan, L. N., Li, B., Wang, W. T., et al. (2017). Transplantation versus hepatectomy for HCC beyond the Milan criteria: A propensity score analysis. *International journal of surgery (London, England)*, *44*, 33–42. https://doi.org/10.1016/j.ijsu.2017.05.034

117. Sogawa H, Shrager B, Jibara G, Tabrizian P, Roayaie S, Schwartz M. Resection or transplant-listing for solitary hepatitis C-associated hepatocellular carcinoma: an intention-to-treat analysis. *HPB (Oxford).* 2013;15(2): 134-141.

118. Sotiropoulos, G. C., Drühe, N., Sgourakis, G., Molmenti, E. P., Beckebaum, S., Baba, H. A., et al. (2009). Liver transplantation, liver resection, and transarterial chemoembolization for hepatocellular carcinoma in cirrhosis: which is the best oncological approach?. *Digestive diseases and sciences*, *54*(10), 2264–2273. https://doi.org/10.1007/s10620-008-0604-4

119. Squires, M. H., 3rd, Hanish, S. I., Fisher, S. B., Garrett, C., Kooby, D. A., Sarmiento, J. M., et al. (2014). Transplant versus resection for the management of hepatocellular carcinoma meeting Milan Criteria in the MELD exception era at a single institution in a UNOS region with short wait times. *Journal of surgical oncology*, *109*(6), 533–541. https://doi.org/10.1002/jso.23531

120. Sung, P. S., Yang, H., Na, G. H., Hwang, S., Kang, D., Jang, J. W., et al. (2017). Long-Term Outcome of Liver Resection Versus Transplantation for Hepatocellular Carcinoma in a Region Where Living Donation is a Main Source. *Annals of transplantation*, *22*, 276–284. https://doi.org/10.12659/aot.904287

121. Tan, K. C., Rela, M., Ryder, S. D., Rizzi, P. M., Karani, J., Portmann, B., et al. (1995). Experience of orthotopic liver transplantation and hepatic resection for hepatocellular carcinoma of less than 8 cm in patients with cirrhosis. *The British journal of surgery*, *82*(2), 253–256. https://doi.org/10.1002/bjs.1800820239

122. Tiao, G. M., Bobey, N., Allen, S., Nieves, N., Alonso, M., Bucuvalas, J., et al. (2005). The current management of hepatoblastoma: a combination of chemotherapy, conventional resection, and liver transplantation. *The Journal of pediatrics*, *146*(2), 204–211. https://doi.org/10.1016/j.jpeds.2004.09.011

123. Vargas, V., Castells, L., Balsells, J., Charco, R., González, A., Margarit, C., et al. (1995). Hepatic resection or orthotopic liver transplant in cirrhotic patients with small hepatocellular carcinoma. *Transplantation proceedings*, *27*(1), 1243–1244.

124. Vennarecci, G., Ettorre, G. M., Antonini, M., Santoro, R., Maritti, M., Tacconi, G., et al. (2007). First-line liver resection and salvage liver transplantation are increasing therapeutic strategies for patients with hepatocellular carcinoma and child a cirrhosis. *Transplantation proceedings*, *39*(6), 1857–1860. https://doi.org/10.1016/j.transproceed.2007.05.073

125. Weimann A, Schlitt HJ, Oldhafer KJ, Hoberg S, Tusch G, Raab R. Is liver transplantation superior to resection in early stage hepatocellular carcinoma? *Transplantation proceedings.* 1999;31(1-2): 500-501.

126. Wu Z, Chen W, Ouyang T, Liu H, Cao L. Management and survival for patients with stage-I hepatocellular carcinoma: An observational study based on SEER database. *Medicine (Baltimore).* 2020;99(41): e22118.

11. Rahman A, Assifi MM, Pedroso FE, Maley WR, Sola JE, Lavu H, et al. Is resection equivalent to transplantation for early cirrhotic patients with hepatocellular carcinoma? A meta-analysis. J Gastrointest Surg. 2012 Oct;16(10):1897-909. doi: 10.1007/s11605-012-1973-8. Epub 2012 Jul 27. PMID: 22836922.

12. Shea BJ, Reeves BC, Wells G, Thuku M, Hamel C, Moran J, et al. AMSTAR 2: a critical appraisal tool for systematic reviews that include randomised or non-randomised studies of healthcare interventions, or both. BMJ. 2017 Sep 21;358:j4008. doi: 10.1136/bmj.j4008. PMID: 28935701; PMCID: PMC5833365.

18. Koh, J. H., Tan, D. J. H., Ong, Y., Lim, W. H., Ng, C. H., Tay, P. W. L., et al. Liver resection versus liver transplantation for hepatocellular carcinoma within Milan criteria: a meta-analysis of 18,421 patients. *Hepatobiliary surgery and nutrition*, *11*(1), 78–93. https://doi.org/10.21037/hbsn-21-350

21. Menahem, B., Lubrano, J., Duvoux, C., Mulliri, A., Alves, A., Costentin, C., et al. Liver transplantation versus liver resection for hepatocellular carcinoma in intention to treat: An attempt to perform an ideal meta-analysis. *Liver transplantation : official publication of the American Association for the Study of Liver Diseases and the International Liver Transplantation Society*, *23*(6), 836–844. https://doi.org/10.1002/lt.24758

23. Schoenberg, M. B., Bucher, J. N., Vater, A., Bazhin, A. V., Hao, J., Guba, M. O., et al. Resection or Transplant in Early Hepatocellular Carcinoma. *Deutsches Arzteblatt international*, *114*(31-32), 519–526. https://doi.org/10.3238/arztebl.2017.0519

25. Zheng, Z., Liang, W., Milgrom, D. P., Zheng, Z., Schroder, P. M., Kong, N. S., et al. Liver transplantation versus liver resection in the treatment of hepatocellular carcinoma: a meta-analysis of observational studies. *Transplantation*, *97*(2), 227–234. https://doi.org/10.1097/TP.0b013e3182a89383

26. Vogel, A., Martinelli, E., ESMO Guidelines Committee. Electronic address: clinicalguidelines@esmo.org, & ESMO Guidelines Committee (2021). Updated treatment recommendations for hepatocellular carcinoma (HCC) from the ESMO Clinical Practice Guidelines. *Annals of oncology : official journal of the European Society for Medical Oncology*, *32*(6), 801–805. https://doi.org/10.1016/j.annonc.2021.02.014

29. Kanneganti, M., Mahmud, N., Kaplan, D. E., Taddei, T. H., & Goldberg, D. S. (2020). Survival Benefit of Liver Transplantation for Hepatocellular Carcinoma. *Transplantation*, *104*(1), 104–112. https://doi.org/10.1097/TP.0000000000002816

31. Viswanathan, M., Ansari, M. T., Berkman, N. D., Chang, S., Hartling, L., McPheeters, M., et al (2012). Assessing the Risk of Bias of Individual Studies in Systematic Reviews of Health Care Interventions. In *Methods Guide for Effectiveness and Comparative Effectiveness Reviews*. Agency for Healthcare Research and Quality (US).

32. Joober R, Schmitz N, Annable L, Boksa P. Publication bias: what are the challenges and can they be overcome? *J Psychiatry Neurosci.* 2012;37(3): 149-152.

35. Adam, R., Azoulay, D., Castaing, D., Eshkenazy, R., Pascal, G., Hashizume, K., et al (2003). Liver resection as a bridge to transplantation for hepatocellular carcinoma on cirrhosis: a reasonable strategy?. *Annals of surgery*, *238*(4), 508–519. https://doi.org/10.1097/01.sla.0000090449.87109.44

36. Adam, R., Bhangui, P., Vibert, E., Azoulay, D., Pelletier, G., Duclos-Vallée, J. C., et al. (2012). Resection or transplantation for early hepatocellular carcinoma in a cirrhotic liver: does size define the best oncological strategy?. *Annals of surgery*, *256*(6), 883–891. https://doi.org/10.1097/SLA.0b013e318273bad0

37. Aksoy, S. O., Unek, T., Sevinc, A. İ., Arslan, B., Sirin, H., Derici, Z. S., et al. (2020). Comparison of Resection and Liver Transplant in Treatment of Hepatocellular Carcinoma. *Experimental and clinical transplantation : official journal of the Middle East Society for Organ Transplantation*, *18*(6), 712–718. https://doi.org/10.6002/ect.2017.0303.

38. Baccarani, U., Isola, M., Adani, G. L., Benzoni, E., Avellini, C., Lorenzin, D., et al. (2008). Superiority of transplantation versus resection for the treatment of small hepatocellular carcinoma. *Transplant international : official journal of the European Society for Organ Transplantation*, *21*(3), 247–254. https://doi.org/10.1111/j.1432-2277.2007.00597.x

39. Belghiti, J., Cortes, A., Abdalla, E. K., Régimbeau, J. M., Prakash, K., Durand, F., Sommacale, D., et al. (2003). Resection prior to liver transplantation for hepatocellular carcinoma. *Annals of surgery*, *238*(6), 885–893. https://doi.org/10.1097/01.sla.0000098621.74851.65

40. Bellavance, E. C., Lumpkins, K. M., Mentha, G., Marques, H. P., Capussotti, L., Pulitano, C., et al. (2008). Surgical management of early-stage hepatocellular carcinoma: resection or transplantation?. *Journal of gastrointestinal surgery : official journal of the Society for Surgery of the Alimentary Tract*, *12*(10), 1699–1708. https://doi.org/10.1007/s11605-008-0652-2

41. Bigourdan, J. M., Jaeck, D., Meyer, N., Meyer, C., Oussoultzoglou, E., Bachellier, P., et al. (2003). Small hepatocellular carcinoma in Child A cirrhotic patients: hepatic resection versus transplantation. *Liver transplantation : official publication of the American Association for the Study of Liver Diseases and the International Liver Transplantation Society*, *9*(5), 513–520. https://doi.org/10.1053/jlts.2003.50070

43. Borie, F., Bouvier, A. M., Herrero, A., Faivre, J., Launoy, G., Delafosse, P., et al. (2008). Treatment and prognosis of hepatocellular carcinoma: a population based study in France. *Journal of surgical oncology*, *98*(7), 505–509. https://doi.org/10.1002/jso.21159

44. Bronowicki, J. P., Boudjema, K., Chone, L., Nisand, G., Bazin, C., Pflumio, F., et al. (1996). Comparison of resection, liver transplantation and transcatheter oily chemoembolization in the treatment of hepatocellular carcinoma. *Journal of hepatology*, *24*(3), 293–300. https://doi.org/10.1016/s0168-8278(96)80007-9

45. Canter, R. J., Patel, S. A., Kennedy, T., D'Angelica, M. I., Jarnagin, W. R., Fong, Y., et al. (2011). Comparative analysis of outcome in patients with hepatocellular carcinoma exceeding the milan criteria treated with liver transplantation versus partial hepatectomy. *American journal of clinical oncology*, *34*(5), 466–471. https://doi.org/10.1097/COC.0b013e3181ec63dd

46. Cha, C. H., Ruo, L., Fong, Y., Jarnagin, W. R., Shia, J., Blumgart, L. H., et al. (2003). Resection of hepatocellular carcinoma in patients otherwise eligible for transplantation. *Annals of surgery*, *238*(3), 315–323. https://doi.org/10.1097/01.sla.0000086548.84705.ef

47. Chan, S. C., Fan, S. T., Chok, K. S., Cheung, T. T., Chan, A. C., Fung, J. Y., et al. (2011). Survival advantage of primary liver transplantation for hepatocellular carcinoma within the up-to-7 criteria with microvascular invasion. *Hepatology international*, *6*(3), 646–656. https://doi.org/10.1007/s12072-011-9318-3

48. Chan, A. C., Chan, S. C., Chok, K. S., Cheung, T. T., Chiu, D. W., Poon, R. T., et al. (2013). Treatment strategy for recurrent hepatocellular carcinoma: salvage transplantation, repeated resection, or radiofrequency ablation?. *Liver transplantation : official publication of the American Association for the Study of Liver Diseases and the International Liver Transplantation Society*, *19*(4), 411–419. https://doi.org/10.1002/lt.23605

49. Choi, G. H., Kim, D. H., Kang, C. M., Kim, K. S., Choi, J. S., Lee, W. J., et al. (2008). Prognostic factors and optimal treatment strategy for intrahepatic nodular recurrence after curative resection of hepatocellular carcinoma. *Annals of surgical oncology*, *15*(2), 618–629. https://doi.org/10.1245/s10434-007-9671-6

51. Chuan, W., Li, C., Wen, T. F., Yan, L. N., Li, B., Liang, G. L., & Li, K. W. (2014). Short-term and long-term outcomes of surgical treatment for HCC within Milan criteria with cirrhotic portal hypertension. *Hepato-gastroenterology*, *61*(136), 2185–2190.

52. Cillo, U., Vitale, A., Brolese, A., Zanus, G., Neri, D., Valmasoni, M., et al. (2007). Partial hepatectomy as first-line treatment for patients with hepatocellular carcinoma. *Journal of surgical oncology*, *95*(3), 213–220. https://doi.org/10.1002/jso.20641

54. Colella, G., Bottelli, R., De Carlis, L., Sansalone, C. V., Rondinara, G. F., Alberti, A., et al. (1998). Hepatocellular carcinoma: comparison between liver transplantation, resective surgery, ethanol injection, and chemoembolization. *Transplant international : official journal of the European Society for Organ Transplantation*, *11 Suppl 1*, S193–S196. https://doi.org/10.1007/s001470050459

55. Dai, Y., Li, C., Wen, T. F., & Yan, L. N. (2014). Comparison of liver resection and transplantation for Child-pugh A cirrhotic patient with very early hepatocellular carcinoma and portal hypertension. *Pakistan journal of medical sciences*, *30*(5), 996–1000. https://doi.org/10.12669/pjms.305.5038

57. De Carlis L, Sammartino C, Giacomoni A. [Surgical treatment of hepatocellular carcinoma: resection or transplantation? Results of a multivariate analysis]. *Chirurgia italiana.* 2001;53(5): 579-586.

58. De Carlis, L., Giacomoni, A., Pirotta, V., Lauterio, A., Slim, A. O., Sammartino, et al. (2003). Surgical treatment of hepatocellular cancer in the era of hepatic transplantation. *Journal of the American College of Surgeons*, *196*(6), 887–897. https://doi.org/10.1016/S1072-7515(03)00140-6

59. Del Gaudio, M., Ercolani, G., Ravaioli, M., Cescon, M., Lauro, A., Vivarelli, M., et al. (2008). Liver transplantation for recurrent hepatocellular carcinoma on cirrhosis after liver resection: University of Bologna experience. *American journal of transplantation : official journal of the American Society of Transplantation and the American Society of Transplant Surgeons*, *8*(6), 1177–1185. https://doi.org/10.1111/j.1600-6143.2008.02229.x

60. Dima, S. O., Iacob, S., Botea, F., Matei, E., Dorobanfu, B., Vasile, S., et al. (2009). Multimodal treatment of hepatocellular carcinoma: an eastern European experience. *Hepato-gastroenterology*, *56*(96), 1696–1703.

61. El-Gazzaz, G., Wong, W., El-Hadary, M. K., Gunson, B. K., Mirza, D. F., et al. (2000). Outcome of liver resection and transplantation for fibrolamellar hepatocellular carcinoma. *Transplant international : official journal of the European Society for Organ Transplantation*, *13 Suppl 1*, S406–S409. https://doi.org/10.1007/s001470050372

62. Facciuto, M. E., Koneru, B., Rocca, J. P., Wolf, D. C., Kim-Schluger, L., Visintainer, P., et al. (2008). Surgical treatment of hepatocellular carcinoma beyond Milan criteria. Results of liver resection, salvage transplantation, and primary liver transplantation. *Annals of surgical oncology*, *15*(5), 1383–1391. https://doi.org/10.1245/s10434-008-9851-z

63. Fan, H. L., Chen, T. W., Hsieh, C. B., Jan, H. C., His, S. C., De-Chuan, C., et al. (2010). Liver transplantation is an alternative treatment of hepatocellular carcinoma beyond the Milan criteria. *American journal of surgery*, *200*(2), 252–257. https://doi.org/10.1016/j.amjsurg.2009.07.049

64. Fan, S. T., Poon, R. T., Yeung, C., Lam, C. M., Lo, C. M., Yuen, W. K., et al. (2011). Outcome after partial hepatectomy for hepatocellular cancer within the Milan criteria. *The British journal of surgery*, *98*(9), 1292–1300. https://doi.org/10.1002/bjs.7583

66. Figueras, J., Jaurrieta, E., Valls, C., Ramos, E., Serrano, T., Rafecas, et al. (2000). Resection or transplantation for hepatocellular carcinoma in cirrhotic patients: outcomes based on indicated treatment strategy. *Journal of the American College of Surgeons*, *190*(5), 580–587. https://doi.org/10.1016/s1072-7515(00)00251-9

67. Foltys, D., Zimmermann, T., Kaths, M., Strempel, M., Heise, M., Hoppe-Lotichius, M., et al. (2014). Hepatocellular carcinoma in Child's A cirrhosis: a retrospective analysis of matched pairs following liver transplantation vs. liver resection according to the intention-to-treat principle. *Clinical transplantation*, *28*(1), 37–46. https://doi.org/10.1111/ctr.12273

68. Franssen, B., Alshebeeb, K., Tabrizian, P., Marti, J., Pierobon, E. S., Lubezky, N., et al. (2014). Differences in surgical outcomes between hepatitis B- and hepatitis C-related hepatocellular carcinoma: a retrospective analysis of a single North American center. *Annals of surgery*, *260*(4), 650–658. https://doi.org/10.1097/SLA.0000000000000917

73. Hsueh, K. C., Lee, T. Y., Kor, C. T., Chen, T. M., Chang, T. M., Yang, S. F., et al. (2016). The role of liver transplantation or resection for patients with early hepatocellular carcinoma. *Tumour biology : the journal of the International Society for Oncodevelopmental Biology and Medicine*, *37*(3), 4193–4201. https://doi.org/10.1007/s13277-015-4243-z

74. Huang, Z. Y., Liang, B. Y., Xiong, M., Dong, K. S., Zhang, Z. Y., Zhang, E. L., et al. (2016). Severity of cirrhosis should determine the operative modality for patients with early hepatocellular carcinoma and compensated liver function. *Surgery*, *159*(2), 621–631. https://doi.org/10.1016/j.surg.2015.09.002

75. Hwang, S., Lee, S. G., Moon, D. B., Ahn, C. S., Kim, K. H., Lee, Y. J., et al. (2007). Salvage living donor liver transplantation after prior liver resection for hepatocellular carcinoma. *Liver transplantation : official publication of the American Association for the Study of Liver Diseases and the International Liver Transplantation Society*, *13*(5), 741–746. https://doi.org/10.1002/lt.21157

76. Iwatsuki, S., Starzl, T. E., Sheahan, D. G., Yokoyama, I., Demetris, A. J., Todo, S., et al. (1991). Hepatic resection versus transplantation for hepatocellular carcinoma. *Annals of surgery*, *214*(3), 221–229. https://doi.org/10.1097/00000658-199109000-00005

78. Kaido, T., Morita, S., Tanaka, S., Ogawa, K., Mori, A., Hatano, E., et al. (2015). Long-term outcomes of hepatic resection versus living donor liver transplantation for hepatocellular carcinoma: a propensity score-matching study. *Disease markers*, *2015*, 425926. https://doi.org/10.1155/2015/425926

80. Koniaris, L. G., Levi, D. M., Pedroso, F. E., Franceschi, D., Tzakis, A. G., Santamaria-Barria, J. A., et al. (2011). Is surgical resection superior to transplantation in the treatment of hepatocellular carcinoma?. *Annals of surgery*, *254*(3), 527–538. https://doi.org/10.1097/SLA.0b013e31822ca66f

81. Kooby, D. A., Egnatashvili, V., Graiser, M., Delman, K. A., Kauh, J., Wood, W. C., et al. (2008). Changing management and outcome of hepatocellular carcinoma: evaluation of 501 patients treated at a single comprehensive center. *Journal of surgical oncology*, *98*(2), 81–88. https://doi.org/10.1002/jso.21049

86. Li, C., Zhu, W. J., Wen, T. F., Dai, Y., Yan, L. N., Li, B., et al. (2014). Child-Pugh A hepatitis B-related cirrhotic patients with a single hepatocellular carcinoma up to 5 cm: liver transplantation vs. resection. *Journal of gastrointestinal surgery : official journal of the Society for Surgery of the Alimentary Tract*, *18*(8), 1469–1476. https://doi.org/10.1007/s11605-014-2550-0

87. Li, C., Liu, J. Y., Peng, W., Wen, T. F., Yan, L. N., Yang, J. Y., et al. (2017). Liver resection *versus* transplantation for multiple hepatocellular carcinoma: a propensity score analysis. *Oncotarget*, *8*(46), 81492–81500. https://doi.org/10.18632/oncotarget.20623

88. Lim, C., Shinkawa, H., Hasegawa, K., Bhangui, P., Salloum, C., Gomez Gavara, C., et al. (2017). Salvage liver transplantation or repeat hepatectomy for recurrent hepatocellular carcinoma: An intent-to-treat analysis. *Liver transplantation : official publication of the American Association for the Study of Liver Diseases and the International Liver Transplantation Society*, *23*(12), 1553–1563. https://doi.org/10.1002/lt.24952

92. Meyerovich, G., Goykhman, Y., Nakache, R., Nachmany, I., Lahat, G., Shibolet, O., et al. (2019). Resection vs Transplant Listing for Hepatocellular Carcinoma: An Intention-to-Treat Analysis. *Transplantation proceedings*, *51*(6), 1867–1873. https://doi.org/10.1016/j.transproceed.2019.02.030

93. Michel, J., Suc, B., Montpeyroux, F., Hachemanne, S., Blanc, P., Domergue, J., et al. (1997). Liver resection or transplantation for hepatocellular carcinoma? Retrospective analysis of 215 patients with cirrhosis. *Journal of hepatology*, *26*(6), 1274–1280. https://doi.org/10.1016/s0168-8278(97)80462-x

94. Michelakos, T., Xourafas, D., Qadan, M., Pieretti-Vanmarcke, R., Cai, L., Patel, M. S., et al. (2019). Hepatocellular Carcinoma in Transplantable Child-Pugh A Cirrhotics: Should Cost Affect Resection vs Transplantation?. *Journal of gastrointestinal surgery : official journal of the Society for Surgery of the Alimentary Tract*, *23*(6), 1135–1142. https://doi.org/10.1007/s11605-018-3946-z

97. Obed, A., Tsui, T. Y., Schnitzbauer, A. A., Obed, M., Schlitt, H. J., Becker, H., et al. (2008). Liver transplantation as curative approach for advanced hepatocellular carcinoma: is it justified?. *Langenbeck's archives of surgery*, *393*(2), 141–147. https://doi.org/10.1007/s00423-007-0250-x

99. Park, M. S., Lee, K. W., Kim, H., Choi, Y. R., Hong, G., Yi, N. J., et al. (2017). Primary Living-donor Liver Transplantation Is Not the Optimal Treatment Choice in Patients With Early Hepatocellular Carcinoma With Poor Tumor Biology. *Transplantation proceedings*, *49*(5), 1103–1108. https://doi.org/10.1016/j.transproceed.2017.03.016

100. Perry, J. F., Charlton, B., Koorey, D. J., Waugh, R. C., Gallagher, P. J., Crawford, M. D., et al. (2007). Outcome of patients with hepatocellular carcinoma referred to a tertiary centre with availability of multiple treatment options including cadaveric liver transplantation. *Liver international : official journal of the International Association for the Study of the Liver*, *27*(9), 1240–1248. https://doi.org/10.1111/j.1478-3231.2007.01569.x

102. Philosophe, B., Greig, P. D., Hemming, A. W., Cattral, M. S., Wanless, I., Rasul, I., et al. (1998). Surgical management of hepatocellular carcinoma: resection or transplantation?. *Journal of gastrointestinal surgery : official journal of the Society for Surgery of the Alimentary Tract*, *2*(1), 21–27. https://doi.org/10.1016/s1091-255x(98)80099-1

106. Ruzzenente, A., Capra, F., Pachera, S., Iacono, C., Piccirillo, G., Lunardi, M., Pet al. (2009). Is liver resection justified in advanced hepatocellular carcinoma? Results of an observational study in 464 patients. *Journal of gastrointestinal surgery : official journal of the Society for Surgery of the Alimentary Tract*, *13*(7), 1313–1320. https://doi.org/10.1007/s11605-009-0903-x

107. Sangro, B., Herráiz, M., Martínez-González, M. A., Bilbao, I., Herrero, I., Beloqui, O., et al. (1998). Prognosis of hepatocellular carcinoma in relation to treatment: a multivariate analysis of 178 patients from a single European institution. *Surgery*, *124*(3), 575–583.

108. Sapisochin, G., Castells, L., Dopazo, C., Bilbao, I., Minguez, B., Lázaro, J. L., et al. (2013). Single HCC in cirrhotic patients: liver resection or liver transplantation? Long-term outcome according to an intention-to-treat basis. *Annals of surgical oncology*, *20*(4), 1194–1202. https://doi.org/10.1245/s10434-012-2655-1

109. Sapisochin, G., Bilbao, I., Balsells, J., Dopazo, C., Caralt, M., Lázaro, J. L., et al. (2010). Optimization of liver transplantation as a treatment of intrahepatic hepatocellular carcinoma recurrence after partial liver resection: experience of a single European series. *World journal of surgery*, *34*(9), 2146–2154. https://doi.org/10.1007/s00268-010-0583-4

110. Scatton, O., Zalinski, S., Terris, B., Lefevre, J. H., Casali, A., Massault, P. P., et al. (2008). Hepatocellular carcinoma developed on compensated cirrhosis: resection as a selection tool for liver transplantation. *Liver transplantation : official publication of the American Association for the Study of Liver Diseases and the International Liver Transplantation Society*, *14*(6), 779–788. https://doi.org/10.1002/lt.21431

111. Seshadri, R. M., Besur, S., Niemeyer, D. J., Templin, M., McKillop, I. H., Swan, R. Z., et al. (2014). Survival analysis of patients with stage I and II hepatocellular carcinoma after a liver transplantation or liver resection. *HPB : the official journal of the International Hepato Pancreato Biliary Association*, *16*(12), 1102–1109. https://doi.org/10.1111/hpb.12300

112. Shabahang, M., Franceschi, D., Yamashiki, N., Reddy, R., Pappas, P. A., Aviles, K., et al. (2002). Comparison of hepatic resection and hepatic transplantation in the treatment of hepatocellular carcinoma among cirrhotic patients. *Annals of surgical oncology*, *9*(9), 881–886. https://doi.org/10.1007/BF02557525

113. Shah, S. A., Cleary, S. P., Tan, J. C., Wei, A. C., Gallinger, S., Grant, D. R., et al. (2007). An analysis of resection vs transplantation for early hepatocellular carcinoma: defining the optimal therapy at a single institution. *Annals of surgical oncology*, *14*(9), 2608–2614. https://doi.org/10.1245/s10434-007-9443-3

116. Shen, J. Y., Li, C., Wen, T. F., Yan, L. N., Li, B., Wang, W. T., et al. (2017). Transplantation versus hepatectomy for HCC beyond the Milan criteria: A propensity score analysis. *International journal of surgery (London, England)*, *44*, 33–42. https://doi.org/10.1016/j.ijsu.2017.05.034

118. Sotiropoulos, G. C., Drühe, N., Sgourakis, G., Molmenti, E. P., Beckebaum, S., Baba, H. A., et al. (2009). Liver transplantation, liver resection, and transarterial chemoembolization for hepatocellular carcinoma in cirrhosis: which is the best oncological approach?. *Digestive diseases and sciences*, *54*(10), 2264–2273. https://doi.org/10.1007/s10620-008-0604-4

119. Squires, M. H., 3rd, Hanish, S. I., Fisher, S. B., Garrett, C., Kooby, D. A., Sarmiento, J. M., et al. (2014). Transplant versus resection for the management of hepatocellular carcinoma meeting Milan Criteria in the MELD exception era at a single institution in a UNOS region with short wait times. *Journal of surgical oncology*, *109*(6), 533–541. https://doi.org/10.1002/jso.23531

120. Sung, P. S., Yang, H., Na, G. H., Hwang, S., Kang, D., Jang, J. W., et al. (2017). Long-Term Outcome of Liver Resection Versus Transplantation for Hepatocellular Carcinoma in a Region Where Living Donation is a Main Source. *Annals of transplantation*, *22*, 276–284. https://doi.org/10.12659/aot.904287

121. Tan, K. C., Rela, M., Ryder, S. D., Rizzi, P. M., Karani, J., Portmann, B., et al. (1995). Experience of orthotopic liver transplantation and hepatic resection for hepatocellular carcinoma of less than 8 cm in patients with cirrhosis. *The British journal of surgery*, *82*(2), 253–256. https://doi.org/10.1002/bjs.1800820239

122. Tiao, G. M., Bobey, N., Allen, S., Nieves, N., Alonso, M., Bucuvalas, J., et al. (2005). The current management of hepatoblastoma: a combination of chemotherapy, conventional resection, and liver transplantation. *The Journal of pediatrics*, *146*(2), 204–211. https://doi.org/10.1016/j.jpeds.2004.09.011

123. Vargas, V., Castells, L., Balsells, J., Charco, R., González, A., Margarit, C., et al. (1995). Hepatic resection or orthotopic liver transplant in cirrhotic patients with small hepatocellular carcinoma. *Transplantation proceedings*, *27*(1), 1243–1244.

124. Vennarecci, G., Ettorre, G. M., Antonini, M., Santoro, R., Maritti, M., Tacconi, G., et al. (2007). First-line liver resection and salvage liver transplantation are increasing therapeutic strategies for patients with hepatocellular carcinoma and child a cirrhosis. *Transplantation proceedings*, *39*(6), 1857–1860. https://doi.org/10.1016/j.transproceed.2007.05.073

127. Yamamoto, J., Iwatsuki, S., Kosuge, T., Dvorchik, I., Shimada, K., Marsh, J. W., et al. (1999). Should hepatomas be treated with hepatic resection or transplantation?. *Cancer*, *86*(7), 1151–1158. https://doi.org/10.1002/(sici)1097-0142(19991001)86:7<1151::aid-cncr8>3.0.co;2-v

128. Yamashita, Y., Yoshida, Y., Kurihara, T., Itoh, S., Harimoto, N., Ikegami, T., et al. (2015). Surgical results for recurrent hepatocellular carcinoma after curative hepatectomy: Repeat hepatectomy versus salvage living donor liver transplantation. *Liver transplantation : official publication of the American Association for the Study of Liver Diseases and the International Liver Transplantation Society*, *21*(7), 961–968. https://doi.org/10.1002/lt.24111

129. Yang, A., Ju, W., Yuan, X., Han, M., Wang, X., Guo, Z., et al. (2017). Comparison between liver resection and liver transplantation on outcomes in patients with solitary hepatocellular carcinoma meeting UNOS criteria: a population-based study of the SEER database. *Oncotarget*, *8*(57), 97428–97438. https://doi.org/10.18632/oncotarget.22134

130. Yokoi, H., Isaji, S., Yamagiwa, K., Tabata, M., Nemoto, A., Sakurai, H., et al. (2006). The role of living-donor liver transplantation in surgical treatment for hepatocellular carcinoma. *Journal of hepato-biliary-pancreatic surgery*, *13*(2), 123–130. https://doi.org/10.1007/s00534-005-1018-8

131. Zaydfudim, V. M., Vachharajani, N., Klintmalm, G. B., Jarnagin, W. R., Hemming, A. W., Doyle, M. B., et al. (2016). Liver Resection and Transplantation for Patients With Hepatocellular Carcinoma Beyond Milan Criteria. *Annals of surgery*, *264*(4), 650–658. https://doi.org/10.1097/SLA.0000000000001866.

132. Zhou, J., Wang, Z., Qiu, S. J., Huang, X. W., Sun, J., Gu, W., et al. (2010). Surgical treatment for early hepatocellular carcinoma: comparison of resection and liver transplantation. *Journal of cancer research and clinical oncology*, *136*(9), 1453–1460. https://doi.org/10.1007/s00432-010-0802-2
